# Retinal biomarkers in schizophrenia spectrum disorders: evidence and implications for the neurodevelopmental and neurodegenerative models

**DOI:** 10.3389/fmed.2025.1697871

**Published:** 2026-01-20

**Authors:** Brittany A. Blose, Steven M. Silverstein

**Affiliations:** 1 Department of Psychology, University of Rochester, Rochester, NY, United States; 2 Department of Psychiatry, University of Rochester Medical Center, Rochester, NY, United States; 3 Department of Ophthalmology, University of Rochester Medical Center, Rochester, NY, United States; 4 Department of Neuroscience, University of Rochester Medical Center, Rochester, NY, United States

**Keywords:** neurodegeneration, neurodevelopment, optical coherence tomography (OCT), retina, schizophrenia

## Abstract

Retinal morphological and functional alterations, such as changes in the thickness and volume of the retinal neural layers, architecture of the microvasculature, and functioning of neurons, have been observed in schizophrenia and have been interpreted in terms of neurodegenerative aspects of the disorder. However, little consideration has been given to the issue of whether, and the extent to which, these retinal differences may reflect neurodevelopmental features of schizophrenia. There are also no current conceptualizations that integrate retinal alteration findings in schizophrenia across different stages of illness, thereby helping to integrate neurodevelopmental and neurodegenerative perspectives on pathophysiology. Therefore, the present review aims to organize evidence of retinal abnormalities in schizophrenia in terms of findings from clinical high-risk for psychosis (CHR), genetic risk, first-episode psychosis (FEP), and chronic schizophrenia samples, and to consider factors such as age and duration of illness. Our goal is to move toward a lifespan model that integrates and transcends prior neurodevelopmental and neurodegenerative viewpoints. Toward this end, we also review studies of retinal alterations among those with prenatal/perinatal insults, neurodevelopmental disorders, and neurological soft signs, as such data can inform what has been observed in schizophrenia. We also mention, where appropriate, relevant findings from neurodegenerative disorders. A better understanding of the trajectories of central nervous system differences throughout the lifespan in people with schizophrenia, as observed in the retina (often called “a window to the brain”), can aid in understanding brain dysfunction in the disorder, assist with characterizing heterogeneity in clinical course, and inform more targeted prevention, monitoring, and intervention efforts.

## Introduction

The earliest view of schizophrenia, conceptualized by Kraepelin (1), was that it is an early-onset form of dementia (“dementia praecox”). This theory has received criticism due to significant heterogeneity in the illness's course and functional outcomes, with many patients not experiencing progressive symptomatic or cognitive impairment or functional decline, even though premorbid levels of functioning are rarely re-attained (2–5). However, about 30%−50% of patients with schizophrenia do experience cumulative residual symptoms, which may be indicative of a progressive trajectory, at least for a proportion of individuals (6–8). Moreover, many patients do experience cognitive decline and progressive gray and white matter loss that exceeds age-related norms, and that is related to poor outcomes (9–12), and the rates of dementia diagnoses in people with schizophrenia are in excess of age norms (13, 14). These data are consistent with both Kraepelin's view and with the recent hypothesis that schizophrenia involves a form of accelerated aging (15–19). In contrast, the neurodevelopmental hypothesis posits that early-life pathological processes (e.g., prenatally) disrupt typical neural development, eventually leading to the manifestation of psychotic illness in adolescence and early adulthood (5, 20–23). According to Lewis and Levitt (24), a genetic predisposition to schizophrenia causes alterations in the expression of genes whose proteins are critical for brain function. These genetic factors interact with environmental factors during ontogenesis or pregnancy (e.g., prenatal infections, exposure to toxins, or chronic stress), leading to cumulative disruptions of developmental processes. In this view, once adolescent development is complete, there are no additional neurobiological consequences beyond those of typical maturation and aging (5, 22). The neurodevelopmental hypothesis can explain features observed in children and adolescents who later go on to develop schizophrenia (25, 26), but it does not fully account for the progressive changes seen in many patients.

Many researchers agree with an integration of the neurodevelopmental and neurodegenerative theories, or the idea that schizophrenia is a “progressive neurodevelopmental disorder” (27). This view has received support from research demonstrating overlap between the genetic and proteomic bases of neurodevelopment and neurodegeneration, that certain genetic and neurodevelopmental disorders are characterized by progressive cognitive decline, and that neurodegenerative disorders share commonalities with aspects of abnormal neural development (28–35).

**Figure 1:**
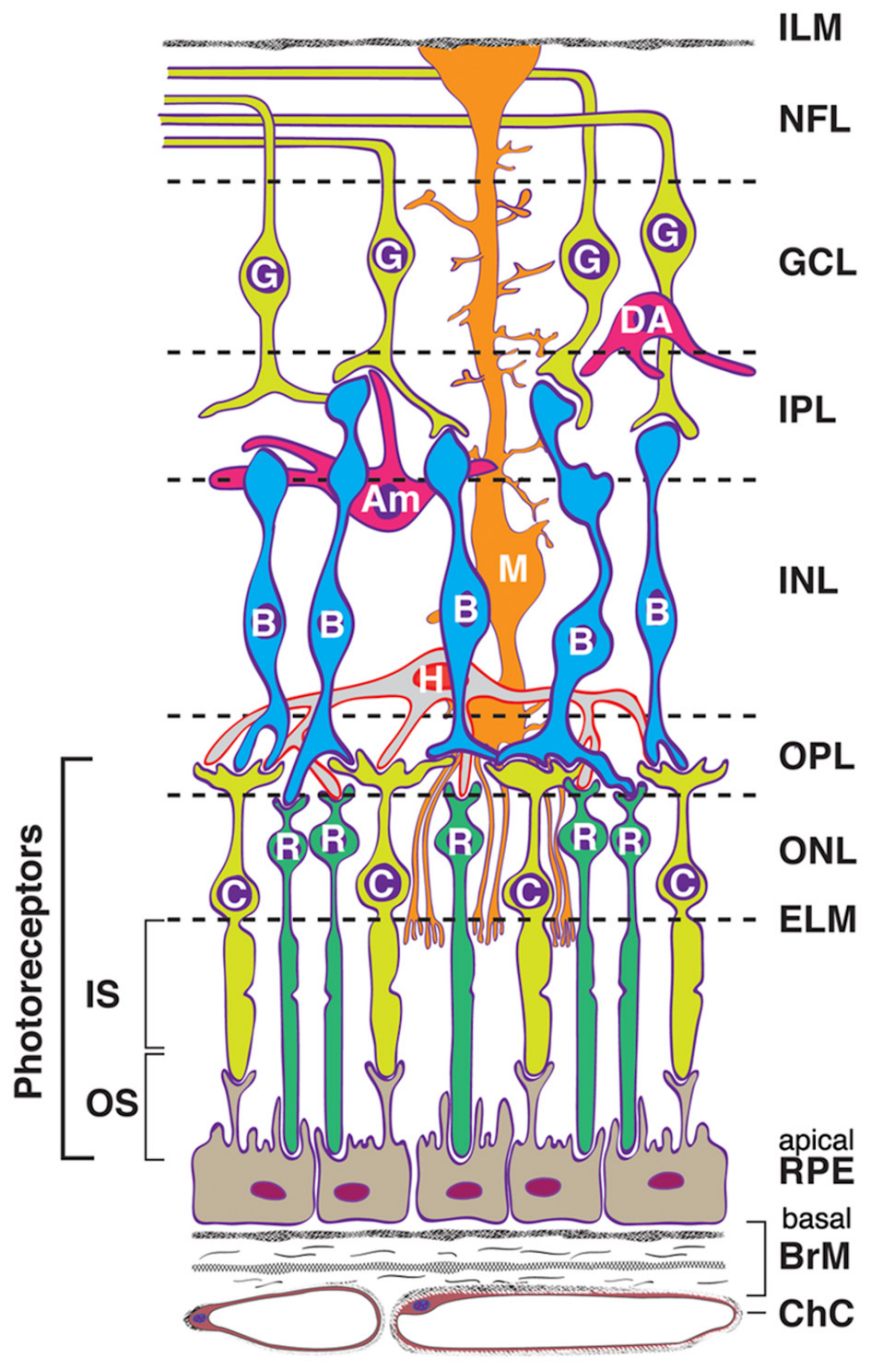
Visual depiction of retinal cell types and layers. Cells: RPE, retinal pigment epithelium (support to photoreceptors); C, cone photoreceptor; R, rod photoreceptor; H, horizontal cell; B, bipolar cell; M, Müller cell (radial glial cell); Am, amacrine cell; DA, displaced amacrine cell; G, ganglion cell (output neuron). Müller cells (M) form the ELM, and their foot processes partially form the ILM. Layers: ChC, choriocapillaris (capillary bed for RPE and photoreceptors); BrM, Bruch's membrane (vessel wall and RPE substratum); ELM, external limiting membrane (junctional complexes); ONL, outer nuclear layer; OPL, outer plexiform layer (synapses); INL, inner nuclear layer; IPL, inner plexiform layer (includes ganglion cell dendrites, bipolar cell axons, and amacrine cells); GCL, ganglion cell (body) layer; NFL, nerve fiber layer (ganglion cell axons); ILM, inner limiting membrane. Reproduced from “Human Eye C, chorioretinal cells and layers” by Wenchao Zheng, Rachel E. Reem, Saida Omarova, Suber Huang, Pier Luigi DiPatre, Casey D. Charvet, Christine A. Curcio and Irina A. Pikuleva, licensed under CC BY 4.0.

**Figure 2:**
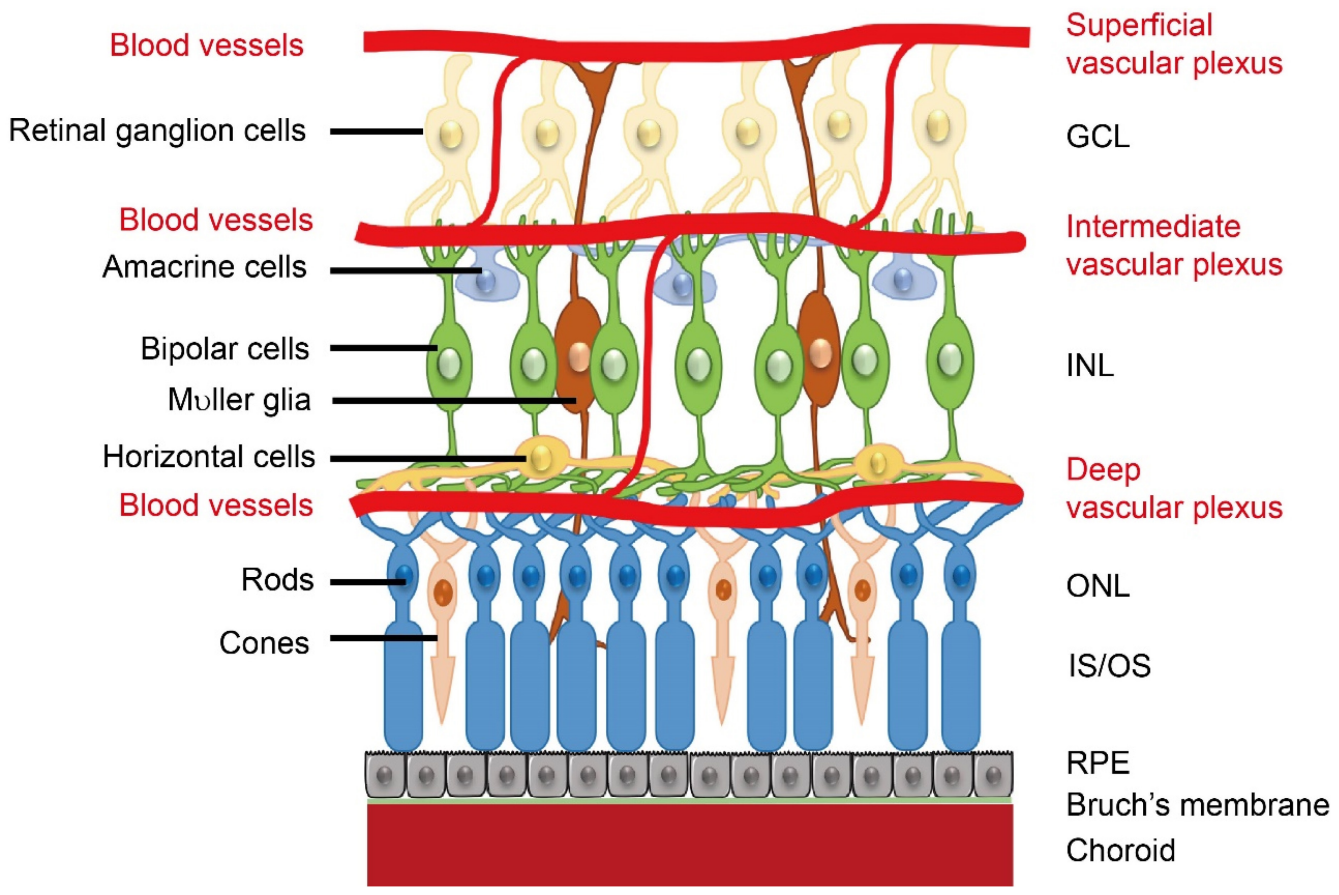
Retinal neural layers and microvasculature plexuses. Retinal microvasculature plexuses are displayed with associated neural layers. GCL, ganglion cell layer; INL, inner nuclear layer; ONL, outer nuclear layer; IS/OS, inner segments/outer segments; RPE, retinal pigment epithelium. Reproduced from “Schematics of retinal neuronal and vascular structure” by Zhongjie Fu, Ye Sun, Bertan Cakir, Yohei Tomita, Shuo Huang, Zhongxiao Wang, Chi-Hsiu Liu, Steve S. Cho, William Britton, Timothy S. Kern, David A. Antonetti, Ann Hellström, and Lois E.H. Smith, licensed under CC BY 4.0.

Biomarker studies examining genetic factors, neural processes, brain structure and function, and neuroinflammatory markers have helped to clarify the etiology of schizophrenia and its neurodevelopmental/neurodegenerative features, but the exact pathophysiological mechanisms remain obscure. More recently, researchers have begun studying retinal structure and function in individuals with schizophrenia to understand these mechanisms better. The retina, which can be thought of as a window to the brain, develops from the same tissue as the brain, the neuroectoderm, and possesses neural characteristics similar to those of the cortex, including neurons, glial cells, and similar neurotransmitters and receptor types (36) (see Figures 1, 2). It also shares the same microvascular and immunological features of the brain (37), and many studies have reported associations between retinal neural/microvascular changes and cognitive decline, decreased brain volume, and brain atrophy in the general population (38–44). There is also robust evidence that retinal morphological alterations are associated with cognitive decline, functional impairment, and disease progression in Alzheimer's disease, Parkinson's disease, and multiple sclerosis (MS) (45–53). Another benefit of studying the retina in this context is that it is much more accessible than the brain, as it is the only part of the central nervous system (CNS) that can be viewed *in vivo*, non-invasively. Further, retinal imaging techniques, such as optical coherence tomography (OCT) and OCT angiography (OCTA), are highly reliable (54), quick, radiation-free, and significantly less expensive than neuroimaging (55), making them ideal methods for retinal biomarker identification that can be integrated into clinical practice to monitor disease risk, course, and progression, which is already occurring in MS (56).

A multitude of studies have now shown that schizophrenia is associated with abnormalities in retinal neural thickness, microvasculature, and cell functioning (57–69). In particular, most of the literature on the retina in schizophrenia has focused on retinal neural structure, revealing in some studies that individuals with schizophrenia have reduced thickness of the retinal nerve fiber layer (RNFL) adjacent to the optic disc, and, more consistently, reduced thickness and volume of the macula, which reflects in large part thinning of the ganglion cell layer and inner plexiform layer (GCL-IPL). Although there are inconsistencies across studies, other layers and characteristics of the retina appear to be affected as well, such as the photoreceptor layer, inner nuclear layer (INL), outer nuclear layer (ONL), outer plexiform layer (OPL), retinal pigment epithelium (RPE), optic cup, and the optic cup-to-disc ratio (70–74) (see Table 1 for a glossary of retinal terms and acronyms). These retinal abnormalities are associated with poorer performance on cognitive assessments, reduced cortical thickness, white matter hypo-intensities, and smaller total brain and white matter volume (70, 75–77). Further, because retinal ganglion cell axons are unmyelinated, RNFL thinning can be considered an unambiguous measure of axonal loss (68, 78). Thus, retinal biomarkers may help elucidate our understanding of the neuropathological mechanisms of schizophrenia and how both neurodevelopmental and neurodegenerative processes are operative in the disorder. Importantly, while retinal findings are not specific to schizophrenia, they are valuable indices of neurodevelopmental and neurodegenerative insults that could be used to identify important clinical outcomes, such as cognitive decline, illness progression, and treatment response.

**Table 1:** Retinal terminology and their definitions.

**Term**	**Definition**
Amacrine cells	A heterogeneous group of interneurons that modulate the input signals from bipolar cells to the ganglion cells (265)
Bipolar cells	Second-order long-projection neurons that project visual input from photoreceptors to retinal ganglion cells and amacrine cells (266, 267)
Choroid	A complex layer of vasculature between the retina and the sclera that plays an important role in supplying oxygen and nourishment to the outer retina (268)
Choroidal vascularity index (CVI)	An index of choroid vascularity, defined as luminal (i.e., total vascular) choroidal area divided by the total choroidal area (191, 192)
Cup-to-disc ratio	The ratio of surface areas between the optic cup to the area of the optic disc (269)
Flash electroretinography (fERG)	A non-invasive technique that uses a light stimulus to measure the electrical activity of the retina
Ellipsoid zone (EZ)	Hyperreflective layer of the retina; Portion of the inner segment of the photoreceptors, immediately adjacent to the junction between photoreceptor inner and outer segments (270)
Fovea	A small depression located in the center of the macula and is responsible for high-acuity vision; it contains a high saturation of cone photoreceptors (271)
Foveal avascular zone (FAZ)	Region surrounding the fovea that does not contain retinal capillaries; the size/shape of the FAZ has been found to be a good indicator of retinal pathology (272)
Fractal dimension	Parameter of retinal microvasculature measuring the complexity and density of the microvascular network (273)
Ganglion cell complex (gcc)	Comprised of the RNFL, GCL, and IPL
Ganglion cell layer-inner plexiform layer (GCL-IPL)	Sum of the two retinal layers (GCL and IPL) containing the retinal ganglion cell bodies and dendrites, bipolar cell axons, and amacrine cells
Inner nuclear layer (INL)	Layer of the retina consisting of the cell bodies of horizontal cells, bipolar cells, amacrine cells, interplexiform neurons, and Müller cells (274)
Macula	Region in the central retina surrounding the fovea containing the highest density of retinal ganglion cells. It is responsible for sharp, clear, central vision (275)
Macular RNFL (mRNFL)	The section of the RNFL measured in the OCT image centered on the fovea
Müller cells	Glial cells of the human retina that play important roles in structural and metabolic functions (276)
Optic cup	Cup-shaped area in the center of the optic disc (277)
Optic disc	Also referred to as the optic nerve head; It is the anterior part of the optic nerve consisting of retinal ganglion cell axons at the point where they begin to leave the eye (278)
Optical coherence tomography (OCT)	A non-invasive retinal imaging technique that uses low-coherence interferometry to create high resolution 2D and 3D images of the retina (55) from which indices such as neural layer thickness and volume can be generated
Optical coherence tomography angiography (OCTA)	A functional extension of OCT that generates high resolution angiograms of the retinal microvasculature (55) from which indices such as perfusion density, vessel length, and foveal avascular zone area can be generated
Outer nuclear layer (ONL)	Layer of the retina containing the cell bodies of the photoreceptor cells (279)
Outer plexiform layer (OPL)	Layer of the retina containing the synapses between photoreceptor cells and interneurons of the INL (280)
Retinal nerve fiber layer (RNFL)	The layer of the retina consisting of retinal ganglion cell axons
Retinal ganglion cells	The output neurons of the retina that project their axons to form the optic nerve and transmit visual data to the brain (281)
Perfusion density	Retinal microvasculature parameter that is often defined as the percentage area of perfused vessels in a region of measurement (282)
Peripapillary RNFL (pRNFL)	The segment of the RNFL measured in the OCT image centered on the optic disc
Photoreceptor layer	Layer of the retina containing the inner and outer segments of the rods and cones (283)
Retinal pigment epithelium (RPE)	The outermost layer of the retina consisting of post-mitotic epithelial cells, which play key roles in the maintenance and survival of the photoreceptors and regulation of the integrity of the choroid capillaries (284)
Retrograde transsynaptic axonal degeneration (RTSD)	Refers to secondary atrophy of pre-synaptic retinal ganglion cells following post-synaptic neuron degeneration in higher-order brain structures (lateral geniculate nucleus in the thalamus) (59, 81, 101)
Tortuosity	Retinal microvascular parameter characterized by abnormal vessel curvature in which the vessels look non-smooth, with more twists and turns (285)

To date, the majority of studies examining retinal alterations in schizophrenia have included individuals with an established, chronic diagnosis. Some of these findings support a neurodegenerative disease process, as retinal layer thinning in these samples becomes more pronounced with longer illness duration beyond the effects of aging (15, 71, 72, 78–83). However, one recent study of patients aged 18–65 years showed that the retinal age gap was most pronounced in the youngest patients and that it declined with advancing age (17). In addition, studies are needed that investigate retinal biomarkers during different phases of the illness, especially the prodromal and early phases of schizophrenia. For example, studying at-risk populations, such as individuals who are at genetic risk due to either a family history of schizophrenia or higher polygenic risk, and those who exhibit preclinical signs and symptoms indicative of a clinical high-risk (CHR) for psychosis syndrome, can enhance our understanding of how schizophrenia develops. Evidence of retinal alterations in these populations would suggest neurodevelopmental origins due to their existence prior to the onset of psychosis. Currently, there is a paucity of research on retinal biomarkers among these individuals; however, there is sufficient evidence to draw tentative conclusions.

Despite the rapidly growing body of evidence for retinal alterations in schizophrenia, and their links to genetic risk for, and clinical features of, schizophrenia, prior reviews of this literature have not integrated the findings into a lifespan view of how schizophrenia develops and progresses over time, even though schizophrenia is, arguably, best conceptualized as a “lifetime disorder,” concerning development, plasticity, and aging (84–88). Therefore, the aim of the current review is to (1) provide an overview of the existing literature on retinal structure findings in schizophrenia^1^ using a life course lens that synthesizes evidence from all phases of the illness, (2) describe the extent to which retinal abnormalities in schizophrenia are consistent with neurodevelopmental and/or neurodegenerative theories, and the extent to which the findings as a whole support the progressive neurodevelopmental hypothesis; and (3) identify gaps in the literature and provide recommendations for future directions in retinal imaging research in this population. While there are also retinal functioning abnormalities in schizophrenia (62), to maintain a focused synthesis of evidence and theory, the current paper will primarily discuss retinal morphological abnormalities for which there is the most evidence.

We will first describe the evidence for the general overlap between neurodevelopmental and neurodegenerative processes to demonstrate how schizophrenia could be characterized by both (i.e., a progressive neurodevelopmental disorder). Next, we will briefly summarize the proposed mechanisms of retinal thinning in schizophrenia. We will then attempt to explain how these retinal findings align with neurodevelopmental and neurodegenerative accounts of schizophrenia using evidence from retinal imaging studies assessing genetic risk, CHR, first-episode psychosis (FEP), and chronic schizophrenia samples. Supplementary Table S1 summarizes the retinal evidence in schizophrenia, noting which findings align with which model. In addition, we will also draw on retinal imaging literature in people exposed to prenatal/perinatal adverse events, neurodevelopmental disorders, and neurodegenerative diseases to further argue these points. Note that this is not meant to be an exhaustive review of the literature, as several meta-analyses and systematic reviews on the topic already exist (57–69), but rather a focused overview of key findings relevant to an integrative lifespan model.

Literature for this review was identified using the keywords schizophrenia, psychosis, clinical-high risk for psychosis, first-episode psychosis, genetic risk for psychosis, optical coherence tomography, retina, and macula on PubMed and Web of Science. Peer-reviewed empirical articles, meta-analyses, and systematic reviews published in English from inception to August 2025 were included.

## Links between neurodevelopmental abnormalities and neurodegeneration

It is increasingly recognized that neurodevelopmental abnormalities increase vulnerability for neurodegeneration later in life, given that both processes share overlapping mechanisms. For example, brain structure development and aging seem to follow a “last in, first out” pattern, in which brain areas that develop later phylogenetically and ontogenetically tend to be the same areas that are most vulnerable to neurodegeneration, such as the association cortices and the neostriatum (28, 89, 90). In support of this theory, Douaud et al. (28) observed a symmetric inverted-U association between age and transmodal cortex gray matter structure variability in a large sample of healthy participants. Because the transmodal cortex develops later than other regions of the brain, and, therefore, may be a strong indicator of “last in, first out” processes, Douaud et al. argue that this finding demonstrates that neurodevelopmental and aging processes mirror each other. Moreover, these regions demonstrated an elevated vulnerability to diseases involving altered neurodevelopment (schizophrenia) and aging (Alzheimer's disease).

Shared molecular mechanisms also link neurodevelopment and neurodegeneration. Similar abnormalities in protein function and protein homeostasis systems, as well as mutations in genes that encode them, have been implicated in both neurodevelopmental and neurodegenerative disorders (29, 30). One example is the function of the amyloid-beta (Aβ) protein and its precursor, amyloid precursor protein (APP), in typical and atypical neurodevelopment (e.g., Down syndrome) and the role they play in neurodegeneration due to Alzheimer's disease. In typical CNS development, APP is involved in proliferation and differentiation of neural stem cells, neuronal migration, neuronal plasticity, and neuronal learning, among other functions, while in the neurodegenerative disease process of Alzheimer's disease, the accumulation of Aβ leads to neuronal damage and neurotransmission dysfunction (30, 91). Overexpression of APP has been found in the placenta of fetuses with Down syndrome and is associated with disruptions in placental development (92). Also, the majority of individuals with Down syndrome show an accumulation of Aβ in their brains by the time they are 40 years of age, which is believed to be related to the high incidence of early-onset dementia due to Alzheimer's disease in this population (30, 93). This suggests that neurodegenerative processes can originate in neurodevelopment, including at the prenatal stage (30).

Neurovascular impairments may be another point of convergence. Impairments in angiogenesis, cerebral blood flow, and the blood-brain barrier have been implicated in the development and progression of both neurodevelopmental disorders (e.g., autism spectrum disorder, schizophrenia, and Down syndrome) and neurodegenerative disorders (e.g., Alzheimer's disease, Huntington's disease, Parkinson's disease, and MS) (33). For example, studies have found an association between decreased cerebral blood flow with increasing age and degenerative changes in schizophrenia (33, 94, 95). In addition, widespread cerebral hypoperfusion has been found in about 75% of children with autism (96) and is associated with language difficulties, executive functioning difficulties, and altered reactivity to sensory input (33). Similarly, reduced cerebral blood flow has been found among individuals with Alzheimer's disease and has been observed before the onset of cognitive decline and plaque deposition (33). The evidence reviewed above, and similar findings, have been used to support the emerging concept that schizophrenia is a progressive neurodevelopmental condition.

## Proposed mechanisms of retinal changes in schizophrenia

Although it is currently unknown which retinal indices are most related to schizophrenia, the evidence so far suggests that macular regions (e.g., GCL-IPL) may be the most sensitive (97). This is thought to be due to the high density of retinal ganglion cells in the macula, as this cell type is highly sensitive to insults (98). The RNFL comprises retinal ganglion cell axons, while the GCL-IPL consists of retinal ganglion cell bodies (GCL) and ganglion cell dendrites, bipolar cell axons, and amacrine cells (IPL). Retinal ganglion cell axons are located parallel to the surface of the retina and converge as the optic nerve, where the RNFL leaves the eye, synapsing onto the lateral geniculate nucleus (LGN) of the thalamus, which relays sensory information to the visual cortex (63, 68, 99).

A question that has yet to be answered is the nature of the relationship between retinal changes and brain changes in schizophrenia. One possibility is that brain changes may lead to retinal structure and function alterations in schizophrenia by the process of retrograde trans-synaptic degeneration (RTSD), which refers to a process involving atrophy of cells in V1 (10, 12, 100), followed by loss of neurons in the LGN that projected to the lost V1 neurons, followed by loss of retinal ganglion cell axons and cell bodies from cells that projected to the lost LGN cells (59, 68, 81, 101). RTSD has been previously demonstrated in MS and occipital lobe injury (102, 103).

On the other hand, findings of retinal changes among individuals with neurodegenerative disorders that become more pronounced with cognitive decline and disease progression (53, 104, 105) suggest that retinal changes and brain changes parallel each other. Evidence for shared genes associated with retinal characteristics, brain MRI traits, eye disorders, and brain disorders (106) also suggests this.

A potential mechanism of concurrent retinal and brain changes is neuroinflammation. It has been proposed that schizophrenia and other psychotic disorders result from an impaired ability to meet the metabolic demands of the brain following disruptions in cerebral blood flow that are caused by genetically mediated inflammatory processes that damage the interconnected system of neurons, astroglia, and microvessels (107–109). At the molecular level, oxidative stress-related gene dysregulation, complement cascade activation, mitochondrial dysfunction, and chronic glial cell activation are believed to result in oxidative damage, disruptions in synaptic pruning, and decreased energy metabolism (110–113). Similar processes also occur in the retina, as chronic inflammation can lead to retinal ganglion cell death through decreased efficiency of the blood-retina barriers, prolonged activation of inflammatory mediators, glutamate excitotoxicity, and oxidative stress (114). For example, oxidative stress genes (e.g., NrF2), complement cascade activation, mitochondrial dysfunction, and chronic glial cell activation have all been implicated in neurodegeneration in both the retina (e.g., age-related macular degeneration, glaucoma, diabetic retinopathy) and the brain (e.g., Alzheimer's disease, Parkinson's disease) (115–121). In schizophrenia, recent studies have revealed associations between inflammatory markers and reduced retinal thickness in patients (82, 122), and a general population study (UK Biobank) indicated that the link between higher polygenic risk scores for schizophrenia and retinal thinning is mediated by genes that also code for neuroinflammatory responses (123).

## Early neurodevelopmental foundations of retinal alterations

### Prenatal and perinatal adverse events and retinal alterations

Prenatal and perinatal insults have long been implicated in the later development of psychosis and are consistent with the neurodevelopmental model of schizophrenia (124, 125). They are also associated with enduring cognitive impairments and neuropsychiatric conditions (126–131). In neuroimaging studies, adults who had prenatal or perinatal risk factors have exhibited similar gray matter alterations and dopaminergic dysfunctions to those seen in schizophrenia, indicating that there might be a disruption to neurodevelopment in the prenatal and/or perinatal period that confers risk for developing schizophrenia (124, 132–134).

Retinal neural layer and microvascular abnormalities (without signs of specific retinal disease), along with atypical brain development, have been observed in people who experienced adverse prenatal and/or perinatal events. Therefore, it is possible that retinal abnormalities in schizophrenia could reflect neurodevelopmental origins. For example, a growing body of research has revealed a range of retinal structural and microvascular abnormalities among infants, children/adolescents, and adults born prematurely (even among those without retinopathy of prematurity) compared to those born full-term (135–144). In addition, evidence suggests that smaller fetal head circumference, lower birth weight, and small-for-gestational-age status are associated with retinal morphological abnormalities (139, 141, 145–149). Moreover, retinal alterations, such as thinner RNFL and GCL-IPL, as well as narrower retinal arteriolar diameter, correlate with poorer cognition and motor skills, as well as smaller brain volumes and more extensive white matter injury, among children and adults with these prenatal/perinatal complications (137, 142, 150–152).

It is believed that reductions in retinal layer thickness among individuals exposed to prenatal and perinatal adverse events may result from disruptions in typical retinal development and suboptimal adaptive mechanisms during gestation, driven by an adverse intrauterine environment (144, 151). Inflammation is another factor proposed to disrupt retinal morphological development concomitantly with prematurity or low birth weight (151). For example, systemic inflammation during the neonatal period can disrupt long-term neuroretinal functioning, as inflammation leads to increased microglial cell activation in the GCL and outer plexiform layer (OPL), resulting in retinal neural cell damage (153, 154).

These findings have important implications for neurodevelopment and schizophrenia. First, OCT findings in people with a history of obstetric complications, and their association with impaired cognition and brain structure, demonstrate that OCT indices are useful indicators of altered neurodevelopment, in addition to growing evidence supporting OCT indices as indicators of neurodegeneration (45–52). Second, because premature birth, low birth weight, and reduced fetal head circumference are associated with abnormal brain anatomy (134, 155–157), poorer cognitive functioning throughout development and into adulthood (158–160), *and are known risk factors for the later development of schizophrenia* (124, 161), this opens the door to the possibility that retinal anomalies in schizophrenia are present perhaps as early as the prenatal period, and reflect alterations in retinal development. What is less clear is whether disruptions in retinal development confer a greater risk for accelerated retinal aging. There is evidence that premature-born adults exhibit accelerated brain aging (162), which suggests that abnormalities in retinal neural and microvascular structure could represent both disturbances in neuroretinal and retinal microvascular development and increased vulnerability to progressive retinal atrophy in schizophrenia.

### Retinal findings in other neurodevelopmental disorders

There is preliminary evidence that retinal abnormalities exist in other neurodevelopmental disorders with overlapping pathophysiology with schizophrenia (163, 164), such as attention-deficit/hyperactivity disorder (ADHD) and autism spectrum disorder (ASD) (165–167). A small body of literature has revealed reduced retinal thickness among children, adolescents, and adults with ADHD, as well as both reduced and increased retinal thickness across the lifespan in ASD. Studies have also found associations between reduced retinal thickness and lower cognitive functioning in people with ASD (168, 169).

Retinal imaging studies of individuals with ADHD and ASD suggest that abnormal retinal morphology may be reflective of atypical neurodevelopment, which suggests that similar mechanisms could occur in schizophrenia. It has been theorized that this may be due to disruptions in neurogenesis and neuronal migration (169) or neuroinflammation from microglia activation (168). Similarly, Friedel et al. (170) hypothesized that retinal volume loss observed in adults with ASD may be due to disturbances in migratory or other neurodevelopmental networks, reflecting simultaneous disturbances in neocortical networks. They also speculate that retinal volume loss may reflect an imbalance of excitatory and inhibitory signals resulting from changes in excitatory glutamatergic projection neurons and inhibitory GABAergic interneurons, which play prominent roles in retinal signal processing (170–172). These changes in excitatory-inhibitory balance are found in the neocortex and hippocampus in both ASD and schizophrenia (173, 174).

## Retinal findings in schizophrenia consistent with the neurodevelopmental theory

### Retinal findings in familial and genetic risk samples

Retinal studies of unaffected first-degree relatives of individuals with schizophrenia have shown differences in retinal neural layer thickness and volume, retinal vessel diameter, and amplitude and latency of flash electroretinography (fERG) waveforms compared to healthy controls (175–181). For example, Kurtulmus et al. (182) found reduced IPL thickness in both chronic, stable schizophrenia patients (mean illness duration = 18.31 years) and their first-degree relatives compared to controls, with no group differences in RNFL, GCL, or macular thickness. In a similar study, Kaya et al. (183) found no group differences in RNFL thickness but observed a graded pattern in GCL-IPL thickness among schizophrenia patients (mean illness duration = 11.97 years), their unaffected siblings, and controls (schizophrenia patients < siblings < controls), although the differences were significant only between the schizophrenia patients and controls. Macular volume showed a different pattern (siblings > controls > schizophrenia patients), with significant differences between the siblings and the schizophrenia patients. Another study reported thinner segments of the peripapillary RNFL (pRNFL, the portion of the RNFL adjacent to the optic disc) and GCL-IPL, as well as reduced macular volume, in schizophrenia patients compared to their unaffected siblings (184), suggesting that unaffected first-degree relatives show genetically driven retinal abnormalities, but that these are less pronounced than those in schizophrenia.

In terms of retinal microvasculature, in a sample of adolescent and young adult monozygotic and dizygotic twins with psychosis symptoms and their unaffected co-twins, Meier et al. (185) found that a higher proportion of probands and their unaffected twins had wider retinal venules relative to controls, which was independent of variables known to influence vasculature, including smoking and body mass index (BMI). Additionally, probands had the widest retinal venules, followed by their twins, and then by controls. This demonstrates that retinal vessel diameter is associated with a familial liability to psychosis symptoms and may not solely represent a consequence of the disease or lifestyle.

Consistent with OCT findings among those with familial risk for schizophrenia, studies using polygenic risk scores for schizophrenia (PRS) and Mendelian randomization have found a relationship between greater genetic risk for schizophrenia and reduced thickness of retinal neural layers (97, 123, 185–188). For example, Blose et al. (97) found that, among 35,024 participants in the UK Biobank, greater polygenic risk was associated with reduced thickness of the GCL-IPL, particularly among those aged 40–59. As noted above, Rabe et al. (123) found that among the genes in the polygenic risk score for schizophrenia related to retinal thickness, there was overlap with genes involved in the neuroinflammatory response. These data indicate specific neurobiological mechanisms by which genetic differences in schizophrenia can affect CNS health (e.g., function of interneurons and neuroinflammation, respectively). They are also consistent with evidence that genetic variants associated with macular thickness overlap with those associated with schizophrenia (189) and that similar genes control retinal and brain characteristics (106, 190).

### Retinal findings in CHR samples

In the first known published OCT study of clinical high risk for psychosis (CHR) individuals (191), researchers observed only an increased choroidal vascularity index (CVI) (192) in CHR and (FEP) groups compared to controls (all between the ages of 12 and 35). However, no differences were found among the three groups on several other choroidal variables measured, suggesting that at the CHR stage, an increase in vascularization may be the only abnormality present. This is consistent with the view noted above that inflammatory and vascular changes may precede neural changes in the development of schizophrenia.

Another study investigating retinal layer thickness and volumes between CHR patients, FEP patients (defined as those who had a single psychotic episode within the last 18 months), and healthy controls (between ages 14 and 31), found increased thickness and/or volume of the macula, GCL, IPL, and inner nuclear layer (INL), in CHR and FEP groups relative to controls, while macular RNFL (mRNFL) thickness and volume were reduced only in the FEP group (193). Interestingly, the CHR and FEP groups did not differ in thickness or volume across any of these indices (except mRNFL volume), suggesting that structural alterations may be present before illness onset. However, the authors hypothesized that the increased thickness and volume observed in the patient groups may be due to neuroinflammation and/or abnormal fluid accumulation resulting from a weakened retinal-blood barrier—mechanisms also proposed to explain null findings of retinal thinning in a sample of patients with a recent psychotic episode (194) and another study of FEP patients with schizophrenia or schizoaffective disorder (195). Ascaso et al. (194) postulated that inflammatory processes may increase retinal thickness, thereby masking axonal damage in schizophrenia. No direct evidence supporting these explanations in schizophrenia exists currently, although increased retinal thickness has been observed during acute episodes of MS (but not in between episodes, when neurodegeneration is visible), which has been attributed to effects of inflammation (196).

Taken together, the findings of these studies suggest that retinal morphological and choroidal vascularity abnormalities may be present before the onset of a psychotic disorder, lending support to the neurodevelopmental hypothesis of schizophrenia. The findings also indicate that in the period immediately before or after the onset of psychotic symptoms, inflammation and other processes related to preservation of neural health (e.g., reactive gliosis) may lead to thickening of neural and vascular aspects of the retina. Over time, however, and with long-term expression of these processes, neural and vascular tissue loss occurs.

## Early-stage retinal indicators bridging neurodevelopmental and neurodegenerative theories

### Retinal findings in FEP samples

Overall, most retinal imaging studies that included FEP samples have found significant differences in retinal parameters between FEP patients with a schizophrenia spectrum disorder and healthy controls (180, 193, 197–204), although one study did not (195). Some studies have observed reduced total retinal thickness and reduction in specific segments/layers, such as the RNFL, macula, and central foveal region, as well as differences in retinal vessel density and fractal dimension among FEP patients with a schizophrenia spectrum disorder compared to controls (193, 197–201), whereas other studies found increased retinal thickness and volume, particularly in the INL, GCL, and IPL (193), as well as in the pRNFL (202). A study comparing OCT indices between FEP patients with schizophrenia (with acute symptoms with a duration of less than 3 months and a total duration of illness of less than 5 years) and age- and sex-matched controls found that macular thickness and pRNFL thickness were reduced in FEP patients relative to controls (although the reduction in pRNFL thickness did not reach statistical significance) (199). Similarly, in the only longitudinal study assessing retinal parameters in schizophrenia, Zhuo et al. (200) found that in a sample of antipsychotic-naïve FEP patients with schizophrenia reporting visual disturbances, baseline retinal thickness and gray matter volume in regions of the visual cortex were reduced compared to controls. Interestingly, after 3 years of treatment with an antipsychotic medication, retinal thickness and gray matter volume in regions of the visual cortex showed significant deterioration in the FEP group, whereas no significant changes were found in the control group. Additionally, the gray matter reduction and retinal thickness reduction rates correlated with increases in self-reported visual disturbances among the FEP patients over the 3-year period. However, after an additional 6-month follow-up period, FEP patients did not exhibit any further deterioration in retinal thickness or in gray matter volume of the visual cortex. These findings suggest that retinal and brain morphological alterations were present early in the disorder and could not have been a result of long-term antipsychotic treatment, although the deterioration found over the initial 3-year period may have been exacerbated by antipsychotic treatment.

When retinal indices of FEP patients have been compared with those of chronic schizophrenia patients, several studies have found differences, including reduced macular thickness and volume, and reduced pRNFL thickness in chronic schizophrenia patients (78, 195, 198, 205). Differences in retinal microvasculature have also been observed (206, 207). In the first study to ever compare OCT parameters between schizophrenia patients in different phases of illness, Lee et al. (78) found that chronic schizophrenia patients (with a duration of illness greater than 2 years and less than 10 years) and chronic schizophrenia patients (with a duration of illness greater than 10 years) had significantly thinner pRNFL compared to acutely ill FEP patients with schizophrenia (with a duration of illness of 2 years or less). Chronic patients (with a duration of illness greater than 10 years) also had significantly reduced average macula thickness compared to the FEP patients. Further, longer duration of illness correlated with reductions in pRNFL thickness, macula thickness, and macula volume. Lee et al. stated that these findings parallel those from neuroimaging studies observing degeneration in brain volume over time among individuals with schizophrenia (208–210) and, therefore, may be reflective of a progressive neurodegenerative disease process.

Some studies, however, have not observed differences in retinal microvascular parameters between FEP and chronic schizophrenia patients (195, 211, 212). For example, one study did not find differences in retinal perfusion density, vessel density, or foveal avascular zone size between a sample of FEP patients and those with later-episode schizophrenia or schizoaffective disorder (212); however, another study comparing these groups within the same sample found significantly reduced macular thickness and volume indices among later-episode, but not first-episode, patients (195). Silverstein et al. hypothesized that these results could indicate that retinal microvascular changes occur earlier than retinal neural changes, consistent with the theory that the pathophysiology of schizophrenia involves neuroinflammatory processes that lead to vascular changes and then to changes in neural structure and function (107, 213).

Overall, the evidence from studies of FEP patients indicates that retinal neural and microvascular alterations are present very early in the disorder (sometimes as reduced tissue levels and sometimes as neuroinflammation-mediated tissue swelling), which may reflect neurodevelopmental and neuroinflammatory processes. Conversely, studies showing significant retinal thickness reduction in chronic schizophrenia samples relative to FEP samples (e.g., FEP patients vs. chronic schizophrenia patients) suggest an additional, neurodegenerative process.

### Retinal findings in relation to neurological soft signs

There is emerging evidence that retinal alterations in schizophrenia may be related to neurological soft signs (NSS) (214), which are indicators of neurodevelopmental abnormalities (215) characterized by subtle neurological impairments in motor coordination, sensory integration, and sequencing of complex motor tasks (216, 217). A recent study by Krukow et al. (214) found that macular thickness, macular volume, GCC, and pRNFL were significantly reduced in a group of schizophrenia inpatients relative to healthy controls, and that greater severity of various NSS was correlated with reductions in these OCT indices (except pRNFL). The researchers argued that these findings reflect a link between neurodevelopmental and neurodegenerative processes in schizophrenia due to the fact that NSS are both risk indicators for the disorder, as they are present before the development of psychosis (consistent with a neurodevelopmental process), and tend to worsen with increasing illness severity and chronicity (218) (consistent with a neurodegenerative process).

## Brief overview of neuroimaging findings in schizophrenia consistent with the neurodegenerative theory

One of the most convincing pieces of evidence in favor of a neurodegenerative disease process in schizophrenia is the finding that individuals with the disorder exhibit accelerated gray matter loss, and to some extent, white matter loss, over time beyond the effects of typical aging (219), although the rate of loss varies across different windows of time (220). Studies have also found that individuals with schizophrenia have a greater brain age gap than healthy controls, which is a parameter estimated from machine learning and neuroimaging data (e.g., cortical thickness and brain volume) representing the difference between a person's model-estimated brain age and chronological age, with higher values reflecting more pronounced accelerated brain aging (221–223). Moreover, brain age gaps, representing deterioration in brain volume and cortical thickness, have been found to increase with longer illness duration, suggesting progressive neurodegeneration (223, 224).

## Retinal findings in schizophrenia consistent with the neurodegenerative theory

### Retinal findings on duration of illness

While there is only one longitudinal retinal imaging study in schizophrenia to date (200), many existing studies have explored the effect of illness duration on retinal morphology, and these studies provide preliminary evidence of retinal degeneration. For example, several studies have found that longer disease durations are associated with reduced retinal and choroid layer thickness and volume (71, 72, 78–83, 186, 205, 207, 214, 225–227). Consistent with this, Celik et al. (80) found that among schizophrenia patients who were either treatment-refractory (mean illness duration = 14.55 years) or treatment-responsive (mean illness duration = 12.04 years), a longer duration of illness and a greater number of hospitalizations were correlated with reduced thickness of the GCL and choroid. Additionally, treatment-refractory patients had significantly reduced GCL volume, IPL volume, and choroid thickness compared to the treatment-responsive patients. These data are consistent with the idea of a Kraepelinian subtype of schizophrenia, one characterized by a more chronic, severe, and deteriorating course of illness with progressive CNS atrophy (228).

One issue worth mentioning is that duration of illness and age are highly correlated, which makes it difficult to disentangle the effects of aging from the relationship, so it is possible that age may be confounding the relationship between longer illness duration and retinal thinning (15). However, some studies have found significant inverse relationships between retinal thickness and duration of illness while statistically controlling for age (71, 79), suggesting that this pattern may not merely be due to the effects of typical aging on the retina. For example, one study found that both first-episode and chronic schizophrenia patients demonstrated a loss of retinal microvasculature that exceeded age-matched controls (206). Interestingly, the vasculature of the first episode group resembled that of the older control group that was matched to the chronic patient group.

### Retinal findings indicative of accelerated aging

There is some evidence that age-related retinal thinning is accelerated in schizophrenia relative to healthy controls (15, 17, 74, 75), which is consistent with research findings of other biomarkers indicating signs of accelerated aging in the disorder (229). For example, Blose et al. (15) found a significant negative relationship between age and retinal thickness, particularly in the GCL-IPL, among individuals with a schizophrenia spectrum disorder. This relationship was significantly more pronounced among individuals with a schizophrenia spectrum disorder than it was in healthy controls. Accelerated retinal thinning with age was also found in the RNFL and macular volume in the schizophrenia spectrum disorder group, although these relationships weakened after accounting for diabetes and hypertension. These findings are consistent with results from a large sample of individuals from the AlzEye project, which found that the rate of GCL-IPL thinning with increasing age was greater for schizophrenia patients than it was for healthy controls (74). Also corroborating these findings, Domagała et al. (75) found that in a group of schizophrenia patients, macular thickness and volume decreased at an accelerated rate relative to a group of healthy controls. They observed that this was particularly apparent among patients aged 32–45 years, relative to those aged 20–31 years and 46–65 years, indicating that accelerated retinal thinning may occur in discrete, narrow intervals, as observed in neuroimaging studies of schizophrenia (220). In partial contrast to these findings, Krukow et al. (17) demonstrated a retinal age gap in schizophrenia, with the largest gaps observed in the youngest patients. This difference may be due to differences in the methods used to calculate the age gap across studies.

In sum, these studies suggest that age-related retinal layer thinning, especially in the macular regions, occurs at a greater rate in schizophrenia compared to those without the disorder, which is consistent with a neuroprogressive disease course. Additionally, accelerated retinal aging may occur across certain windows of time, increasing from middle age to older age. However, these cross-sectional findings need to be corroborated with longitudinal research.

## Discussion

In sum, research using retinal imaging in populations as diverse as schizophrenia, people at high risk for the development of schizophrenia, neurodevelopmental disorders, people with a history of prenatal and perinatal adverse events, and neurodegenerative disorders suggests that retinal imaging findings in schizophrenia are likely to reflect both neurodevelopmental and neurodegenerative aspects of the disease, supporting the progressive neurodevelopmental theory. Specifically, evidence of retinal alterations in genetic risk, familial risk, CHR, and FEP populations is in line with a neurodevelopmental disease process because it implies that retinal alterations are present before the full manifestation of psychotic illness or (in the case of FEP) very early in the illness course, perhaps due to some early life insult disrupting retinal neural development. In addition, findings of retinal thinning in other neurodevelopmental disorders that have genetic and pathophysiological overlap with schizophrenia (e.g., ASD), as well as among individuals exposed to prenatal and perinatal complications, which are known risk factors for schizophrenia (e.g., low birth weight, hypoxia), are indirect evidence consistent with the neurodevelopmental model. Conversely, findings of greater reductions in retinal thickness among chronic schizophrenia patients vs. FEP patients, greater reductions in retinal thickness with increasing illness duration in correlation analyses, and more pronounced age-related retinal thinning among schizophrenia patients relative to controls point to neurodegenerative disease processes.

The idea that retinal abnormalities in schizophrenia reflect both neurodevelopmental and neurodegenerative processes is supported by evidence of overlap in signaling pathways utilized by retinal cells for both development and degeneration (172), such as the Wnt signaling pathway (230) and the Notch signaling pathway (231). It is also consistent with growing evidence that early disruptions in neurodevelopment increase vulnerability for neurodegeneration, given that both are associated with alterations in similar brain regions and impairments in the neurovascular system. Finally, this view is consistent with the finding that alterations in protein processing, tracking, and aggregation during brain development are implicated in neurodegeneration in Alzheimer's disease and Parkinson's disease, and genes that mediate vulnerability to these disorders are associated with neurodevelopmental disorders, such as epilepsy, ASD, and schizophrenia (30).

Although more research is needed, converging evidence tentatively implies that in schizophrenia, retinal degeneration may be the product of disruptions in retinal neural development that can be observed as early as the prodromal phase, and that accelerate as the illness progresses, at least for a significant proportion of individuals with the disorder. Therefore, the extant evidence of retinal findings supports the progressive neurodevelopmental theory of schizophrenia, at least for a subtype of schizophrenia patients.

## Future directions and conclusions

There are several gaps in the retinal imaging in schizophrenia literature that, if addressed, are likely to further elucidate the neurodevelopmental and neurodegenerative trajectories of the condition. First, there is an obvious need for more research examining the retina in young individuals with a CHR syndrome and those at genetic risk for schizophrenia to replicate findings of the small number of existing studies. Studies with these populations are beneficial because these individuals tend to be relatively free from many of the confounding factors typically associated with chronic psychotic disorders, such as long-term antipsychotic medication use, unhealthy lifestyle, and systemic diseases.

In addition, more studies are needed that investigate group differences between at-risk patients, early-onset schizophrenia patients, FEP patients, and chronic schizophrenia patients. Currently, only a few studies have taken this approach (180, 191, 193, 195, 198), so replication is needed to draw definitive conclusions. Doing so can further our understanding of the extent and rate of retinal thinning across different stages of illness. Relatedly, more studies that compare the relationship between age and retinal thickness and volume changes between individuals with schizophrenia and healthy controls are needed to help clarify how retinal atrophy in schizophrenia may be indicative of accelerated aging.

Recently, researchers have developed the retinal age gap index, which, similar to the brain age gap, represents the difference between estimated retinal age derived from retinal imaging data and chronological age using a machine learning model (232). So far, greater retinal age gap has predicted an increased risk for Parkinson's disease, all-cause mortality, mortality due to cardiovascular disease and cancer, more progressive diabetic retinopathy, and stroke, among several other conditions, similar to the brain age gap (233–237). Thus, it will be worthwhile to determine the retinal age gap with CHR, FEP, and chronic schizophrenia patients to predict risk of disease onset (for CHR) and trajectories of progressive neurodegeneration. In addition, the retinal age gap could be used to predict and monitor systemic comorbidities that are significantly more prevalent in schizophrenia and contribute to their earlier mortality (238), which is needed because conditions such as diabetes and cardiovascular disease are not well monitored in this population (239). Further, the data collection methods on which retinal age gap models are based are non-invasive, less expensive, and more accessible than other biomarker methodologies, and well-suited for large datasets (232, 240, 241), making them easier to integrate into clinical care.

The evidence base on retinal imaging in schizophrenia would also benefit from longitudinal studies, as they can help identify the degree to which retinal changes in schizophrenia align with those that occur with typical neurodevelopment and aging, thus aiding our understanding of the neurodevelopmental and neurodegenerative characteristics of the disease. Additionally, longitudinal designs should be employed to investigate how retinal changes across development, maturation, and aging compare with those in the brain over time in schizophrenia, since it is not entirely clear whether patterns of retinal degeneration mirror those of brain degeneration over time. Relatedly, more mixed-method studies that incorporate brain imaging and retinal imaging in schizophrenia are needed to further elucidate the nature of their relationship. If retinal imaging can serve as a proxy for brain imaging, this could have important clinical implications because retinal imaging, such as OCT, is significantly less expensive, quicker, better tolerated by patients, and has fewer exclusion criteria (e.g., weight, metal in body) compared to brain imaging techniques, such as MRI (55).

Lastly, the influence of potential confounders, such as antipsychotic medication, systemic diseases (e.g., hypertension, diabetes, etc.), smoking, BMI, and ocular conditions (e.g., myopia, glaucoma, etc.) on retinal findings in schizophrenia is unclear. Findings in genetic-risk, CHR, and antipsychotic-naïve FEP groups suggest these abnormalities are not secondary to antipsychotic medication. However, as noted by Komatsu et al. (61), it is possible that antipsychotics could affect the retina, given that their primary targets (dopamine D2 receptors) are expressed in the retina (242, 243). There is no direct evidence, though, that antipsychotics cross the blood-retina barrier (61). Studies reporting possible GCL-IPL thinning with antipsychotic use are inconsistent, likely due to medication heterogeneity in terms of their receptor activities, and many studies of chronic patients fail to find a significant association with antipsychotic dose (61). Therefore, additional studies evaluating the effect of these medications on retina structure and function in schizophrenia are warranted.

Research suggests that tobacco smoking is associated with alterations in retinal layer thickness and microvasculature (244, 245), likely via endothelial dysfunction, inflammation, and oxidative stress (246–248). However, its influence on retinal findings in schizophrenia remains unclear because most studies have not measured or controlled for smoking. Recent meta-analyses have generally not found a significant effect of smoking, but a number of studies were not included due to a lack of data on the issue (57, 62, 66, 67). Thus, it will be important for future retinal imaging studies to measure tobacco use in schizophrenia to determine the extent to which it accounts for variance in retinal alterations.

While the effect of systemic diseases and ocular conditions known to affect the retina on retinal imaging findings in schizophrenia remains in question, mounting evidence suggests that retinal thinning in the disorder occurs independently of these factors. Most studies have excluded participants with ocular conditions, and many have also controlled for systemic diseases. For example, a recent meta-analysis, which included only studies that excluded individuals with systemic diseases, revealed significant retinal thinning across most OCT parameters in schizophrenia (62). Similarly, large population-based data show thinner mGCL-IPL and larger cup-to-disc ratio in schizophrenia after adjusting for hypertension and diabetes (74). Microvascular findings may be at least partially attributable to cardiometabolic disease (74), though evidence remains limited due to fewer studies. The influence of BMI on retinal findings in schizophrenia also deserves attention, as obesity is more prevalent in schizophrenia (249, 250) and is generally associated with retinal alterations (251–254), yet most studies have not controlled for these factors. However, those that do have still observed differences in retinal neural layers and microvasculature (122, 183, 198, 255, 256).

One important limitation is that the current paper primarily focuses on structural retinal findings in schizophrenia. Findings on retinal function and physiology using methods such as ERG and pupillometry in schizophrenia are so far consistent with structural findings. For example, there is growing evidence of atypical retinal cell functioning using ERG in schizophrenia, as well as those with familial/genetic risk for schizophrenia and CHR individuals (175, 176, 178, 257–259). In addition, atypical pupil reactivity, such as reduced pupil dilation, has been observed in schizophrenia (260–263), and there is some evidence that larger tonic pupil size in infancy is associated with increased genetic risk for schizophrenia (264). Therefore, future research examining the extent to which functional visual physiological measures, such as ERG and pupillometry, support retinal structure findings in schizophrenia and inform the neurodevelopmental and neurodegenerative models is warranted. Lastly, another limitation is that, because this is a narrative review, it is important to keep in mind the potential influence of selection bias and lack of reproducibility inherent to this type of approach.

In conclusion, schizophrenia is a heterogeneous syndrome possessing both neurodevelopmental and neurodegenerative characteristics, with the extent of evidence for these varying across patients. Retinal imaging in schizophrenia has helped elucidate the neurodegenerative nature of the disease thus far and is now beginning to uncover its neurodevelopmental aspects. Continued research using inexpensive, non-invasive, and rapid data acquisition methodologies, such as OCT, that employ longitudinal designs, group patients according to their illness phase (e.g., CHR vs. FEP vs. chronic), and utilize the retinal age gap index can further our understanding of the pathophysiology of schizophrenia, which can then inform more targeted prevention, treatment, and monitoring strategies.

## Footnotes

1 To reduce verbiage, the current paper will refer to schizophrenia spectrum disorders (encompassing schizophrenia, schizoaffective disorder, schizophreniform disorder, and unspecified psychosis not due to a substance or known physiological condition) as ‘schizophrenia.'
