# Supplementary material for: Retinal biomarkers in schizophrenia spectrum disorders: evidence and implications for the neurodevelopmental and neurodegenerative models
Source: Front Med (Lausanne). 2026 Jan 20;12:1697871. doi: 10.3389/fmed.2025.1697871 (PMC12866618; doi:10.3389/fmed.2025.1697871)
Supplement: Supplementary file 1 [file Table_1.docx]

**Retinal Biomarkers in Schizophrenia Spectrum Disorders: Evidence and Implications for the Neurodevelopmental and Neurodegenerative Models**

Brittany A. Blose & Steven M. Silverstein

**Supplementary Information**

**Supplementary Table 1 (S1): Retinal imaging study characteristics grouped by supportive schizophrenia theory**

| **Study** | **Participant Characteristics** | **Mean Age (SD)^a^** | **Mean Illness Duration (SD)^a^** | **Device** | **Confounders Addressed** | **Findings** | **Supporting Theory** |
| --- | --- | --- | --- | --- | --- | --- | --- |
| Akin et al. (2024) (1) | 28 SZ  28 siblings of SZ  28 controls | 34.61 (9.39)  31.5 (9.56)  33.82 (8.8) | 15.39 (10.10) | SD-OCT (Cirrus HD-OCT 5000) | Excluded individuals with any previous or concurrent systemic disease that could affect the eyes (including diseases, hyperlipidemia, hypertension, and cardiovascular diseases); any prior retinal or ocular pathology (e.g., diabetic retinopathy, uveitis, glaucoma, cataracts, or macular degeneration); a history of ocular surgery or trauma; any neurological condition known to affect eyesight; glaucoma or myopia/hypermetropia (±4  D); substance or alcohol abuse; psychiatric comorbidities; neurodevelopmental disorder; or neurodegenerative disorder  Groups were matched on age, gender, smoking status, and level of education | No group differences in macular thickness parameters  Cup volume was larger in SZ and siblings compared to controls  SZ and siblings had reduced GCL-IPL thickness compared to controls  No significant differences in OCT parameters found between SZ and siblings  Facial Emotion Identification Test (2) scores were positively correlated with GCL-IPL thickness in SZ (but not in siblings or controls) | Neurodevelopmental |
| Bannai et al. (2022) (3) | 30 SSD (SZ and SZA; n=8 early-course and n=15 chronic)  22 controls | 37.0 (12.7)  38.0 (11.6) | 13.63 (12.76) | SS-OCT (Topcon Triton SS-OCT) | Excluded individuals with substance dependence/abuse within the past 6 months; glaucoma, macular  degeneration, retinal occlusions, ocular trauma, or myopia >4.0 D; current pregnancy/breast feeding; head injury with neurological sequelae; intellectual disability; or a history of neurologic disorders  Groups were matched on age, sex, race, BCVA, systolic/diastolic BP, and cardiometabolic disease status | SSD had greater VD and SVD compared to controls  Early-course SSD had higher SVD, FD, and VD compared to controls  No significant differences between controls and chronic SSD nor between early-course and chronic SSD on microvascular indices  More severe positive symptoms (PANSS) were associated with lower superficial VD, primarily in chronic SSD  Greater superficial VD was associated with better global functioning in chronic SSD, while it was associated with worse functioning in early-course SSD  Deep VD, SVD, and FD were positively associated with cognitive functioning in SSD, while it was inversely correlated in controls | Neurodevelopmental |
| Bagcı et al. (2025) (4) | 22 SZ  18 FEP  29 healthy siblings of SZ  31 controls | 15.7^[[1]](#footnote-1)^  15.7^1^  15.2^1^  15.8^1^ | Combined patient sample: 0.83^1^ (1.67)^[[2]](#footnote-2)^  SZ: 1.67^1^ (1.81)^2^  FEP: 0.33^1^ (0.46)^2^ | SD-OCT (Spectralis OCT) | Excluded individuals with refractive error including SPH >±4 D and CYL (astigmatism) > ±1D; BCVA ≥20/25; a history of ocular diseases (including ocular surgery, uveitis, any congenital or acquired optic nerve diseases); vascular retinal diseases; or image quality < 7/10  Groups were matched on age and gender  No participants reported tobacco or illicit substance use | FEP had increased mRNFL thickness compared to siblings of SZ and controls  SZ had increased nasal mRNFL thickness compared to siblings of SZ  FEP had increased GCL thickness compared to siblings of SZ and controls  SZ and their siblings had reduced inferior inner and temporal inner GCL thickness compared to controls  SZ and their siblings had reduced IPL thickness compared to controls  FEP had reduced inferior inner and temporal inner IPL thickness compared to siblings of SZ | Neurodevelopmental |
| Boudriot et al. (2025) (5) | 36,349 participants from the UK Biobank | 57.5 (8.0) | N/A | SD-OCT (Topcon 3D-OCT 1000 Mark II) | Excluded individuals with antipsychotic medication use; diabetes-related eye disorders; other ophthalmological conditions (e.g., glaucoma, macular degeneration, etc.); ICD-10 diagnoses of SZ, schizotypal, or delusional disorders (F20-F29); or missing data on variables of interest or covariates  Statistically controlled for age, sex, OCT image quality, BMI, tobacco smoking status, hypertension, and genotyping array | Genetic risk for schizophrenia was concentrated in genes with specific expression in amacrine cells, which was also found in fetal retinal tissue  Genetic association between horizontal cells, retinal ganglion cells, and bipolar cells and SZ found (although less robust association compared to amacrine cells)  Higher polygenic risk for SZ was associated with reduced GCL-IPL thickness | Neurodevelopmental |
| Demirlek et al. (2023) (6) | 31 FEP  35 CHR  30 controls | 21.2 (4.4)  20.4 (3.6)  21.7 (4.1) | Not reported | SD-OCT (Spectralis OCT) | Excluded individuals with any retinal/chorioretinal disease possibly confounding the ophthalmological assessment; insufficient fixation to allow high-quality imaging; any intraocular media opacities; history of any trauma, ocular surgery or other neurological disorders; any systemic disease which could affect the choroid (e.g. diabetes mellitus, hypertension, cardiac diseases, hematologic diseases); BMI ≥35; myopia > 5 SPH D  or 3 CYL D; hyperopia > 5 D and axial length > 25.5 mm; poor image quality; any inflammatory diseases within the last 8 weeks; substance-induced psychosis; intellectual disability; or other developmental abnormalities (e.g. 22q11.2 deletion, Down syndromes)  Groups were matched on age, gender, and smoking status | FEP had increased CVI and LCA/SCA ratio compared to CHR and controls; no significant difference in CVI or LCA/SCA ratio found between CHR and controls  No significant correlations found between CVI and premorbid ability scores, CPZ, DUP, or symptom severity measures in FEP or CHR | Neurodevelopmental |
| Demirlek et al. (2024) (7) | 30 FEP  34 CHR  28 controls | 21.06 (4.34)  20.44 (3.66)  21.50 (4.11) | 0.38 (0.30) | SD-OCT (Spectralis OCT) | Excluded individuals with any retinal or choroidal disease that could confound the ophthalmological assessment; myopia > 5 SPH D or 3 CYL D; hyperopia > 5 D and axial length > 25.5 mm; insufficient fixation for high-quality imaging; any intraocular media opacities; poor image quality; history of trauma, ocular surgery, or other neurological disorders; intellectual disability; developmental abnormalities (e.g., Down syndrome, DiGeorge syndrome); systemic diseases that could affect the retina (e.g., diabetes mellitus, cardiovascular diseases, hypertension, inflammatory, hematologic diseases); any infectious diseases within the last 8 weeks; BMI ≥35; substance abuse; hearing issues that could disrupt neuropsychological assessments; or current gestation/lactation  Groups were matched on age, gender, level of education, BMI, and smoking status | Total retinal macular thickness and volume were increased in FEP and CHR compared to controls  mRNFL thickness and volume were reduced in FEP compared to controls  GCL, IPL, and INL thickness and volume were increased in FEP compared to controls  GCL volume and INL thickness and volume were increased in CHR compared to controls | Neurodevelopmental |
| Fuyi et al. (2024)^[[3]](#footnote-3)^ (8) | 5562 SZ  208,674 controls | N/A | Not reported | N/A | Excluded SNPs with linkage disequilibrium | RNFL thickness was positively associated with SZ | Neurodevelopmental |
| González-Díaz et al. (2025) (9) | 26 FEP (SZ and SZA)  25 controls | 31.9 (1.25)  32.69 (1.91) | <5 years | SD-OCT (Spectralis OCT) | Excluded individuals with neurological diseases; severe ocular or head trauma; alcohol/substance abuse; hypertension; diabetes; ocular surgery; current pregnancy or breastfeeding; any pre-existing ophthalmological conditions that could affect retinal measurements (e.g., refraction errors >±6 D, macular degeneration, amblyopia, media opacities [i.e., cataracts],  or glaucoma); or age < 18 years, or age > 40 years  Statistically controlled for age, sex, duration of untreated psychosis, PANSS total score, and antipsychotic dose  Groups were matched on age, sex, marital status, alcohol use, and mean BP | FEP had increased pRNFL thickness (but not macular volume, macular thickness, or GCL-IPL thickness) compared to controls  pRNFL thickness was significantly negatively correlated with cognitive functioning | Neurodevelopmental |
| Huang et al. (2020) (10) | 100 untreated FEP SZ  100 controls | 23.0 (2.5)  23.5 (3.0) | 0.21 (0.06) | SD-OCT (Cirrus 4000 HD-OCT) | Excluded individuals with substance abuse; diabetes; HbA1c outside normal range; ophthalmic disease or concurrent OCT-related condition; concurrent systemic (e.g., respiratory, cardiovascular, endocrine, neurologic, liver, or kidney disease) or chronic disease; a history of severe head trauma; current ECT; high-grade myopia (≥600 D); IQ <80; or age <18 or age >35 years  Groups were matched on age, gender, and level of education | 67 FEP patients reported visual perception impairments, and of those 67 FEP, 52 had retinal thickness impairment  FEP had significantly reduced macular thickness compared to controls  Macular thickness was unrelated to PANSS scores | Neurodevelopmental |
| Kaya et al. (2022) (11) | 46 SZ  46 healthy siblings of SZ participants (HS)  46 controls | 31.54 (8.09)  30.35 (8.98)  31.96 (7.97) | 11.97 (6.89) | SD-OCT (Optopol REVO OCT) | Excluded individuals with ophthalmologic diseases; diseases affecting the retina (e.g., any type of diabetes mellitus, hypertension, epilepsy, or history of serious head injury); or substance or alcohol use disorders  Statistically controlled for age, gender, educational status, smoking status and BMI  Groups were matched on age, gender, years of education, and smoking status | GCL-IPL was reduced in SZ compared to HS and controls, but the difference was only significant between SZ and controls  Macula volume was reduced in SZ compared to HS  No significant difference in macula volume between HS and controls  Increased number of hospitalizations and longer illness duration were associated with increased pRNFL nasal thickness  Longer duration of untreated psychosis was associated with reduced pRNFL thickness  Number of antipsychotics and cigarettes, as well as BMI and age were positively correlated with pRNFL thickness | Neurodevelopmental |
| Kurtulmus et al. (2020) (12) | 38 chronic, stable SZ  38 unaffected first-degree relatives of SZ participants  38 controls | 41.08 (11.26)  42.11 (13.88)  38.47 (11.21) | 18.31 (9.74) | SD-OCT (Spectralis OCT) | Excluded participants with a history of or concurrent systemic disease that may involve the eyes (including diabetes, hyperlipidemia, hypertension, and cardiovascular diseases); any history of retinal or ocular pathology, surgery, or trauma (e.g., glaucoma, cataract, macular degeneration, diabetic retinopathy and uveitis); any neurologic condition known to affect the visual pathway; refractive error <−4 SPH D or >+3 SPH D; or substance or alcohol misuse within the past year  Statistically controlled for smoking status, comorbid medical diseases, and BMI  Groups were matched on age, gender, smoking status, BMI, and comorbid medical disease status | No significant group differences found in pRNFL, GCL, or macular thickness  Both SZ and their relatives had reduced IPL thickness compared to controls; SZ and their relatives did not significantly differ in IPL thickness | Neurodevelopmental |
| Meier et al. (2015) (13) | 45 probands with ≥1 symptom of psychosis  24 unaffected co-twins  462 controls  Participants were members of the Brisbane Longitudinal Twin Study (14, 15) | 19.91 (3.09)  19.67 (3.0)  20.74 (3.56) | N/A | Nidek 3-Dx/F fundus camera | Statistically controlled for age, sex, axial length, sphericity, smoking status and BMI | Probands had wider retinal venules than controls; unaffected co-twins had venular diameters that were intermediate between probands and controls  No significant differences found in arteriolar diameter between probands, unaffected co-twins, and controls | Neurodevelopmental |
| Nandan et al. (2025) | 32 unmedicated PSD (affective and non-affective psychosis) FEP  30 controls | 31.66 (8.86)  31.03 (7.41) | 0.6 (0.70) | SD-OCT (Cirrus HD-OCT) | Excluded participants who were <18 years old or >45 years old; drug free for <4 weeks for oral and 12 weeks for depot medications; also excluded those with illness duration >2 years; history of neurological illness; significant head injury; comorbid substance dependence (excluding nicotine and caffeine); other psychiatric disorders; hypertension; diabetes; SPH >/=+6 or </=-6 D; or disorders that could affect retinal structures  Groups were matched on age, sex, education, and tobacco use | FEP had reduced macula volume, as well as reduced GCC, macula CSF, and average macula thickness  Poorer performance on visual memory/visuospatial perception and facial emotion recognition tasks was associated with reduced macular thickness  Discriminant function analysis differentiated FEP from controls with 85.5% accuracy based on a combination of facial emotion recognition and macular thickness | Neurodevelopmental |
| Sarkar et al. (2021) (16) | 20 FEP SZ (presenting with acute symptoms of duration < 3 months and a total illness duration < 5 years)  45% of FEP SZ participants had a family history of a SZ-like illness  20 controls | 28.6 (6.3)  30.25 (9.22) | 2.52 (0.87) | SD-OCT (Spectral OCT/SLO version 4.0, combination imaging system) | Excluded individuals with associated systemic comorbidities (e.g., diabetes, hypertension, and coronary artery disease); ophthalmologic conditions/diseases/traumas/surgeries; refractive error >2 SPH D; significant media opacities precluding good imaging by OCT; or chlorpromazine or thioridazine medication use  Groups were matched on age, gender, and sociodemographic profiles | pRNFL and macular thickness were reduced in FEP SZ compared to controls | Neurodevelopmental |
| Silverstein et al. (2021) (17) | 28 SSD (SZ or SZA; n=12 FEP; n=16 chronic SSD)  37 controls | 32.32 (11.27)  32.22 (12.63) | Chronic SSD: >2 years since first psychotic episode  FEP: <2 years since first psychotic episode | SD-OCT (Cirrus HD-OCT 5000) | Excluded individuals with a history of ophthalmologic injury/disease; neurologic, intellectual, mood, or developmental disorders; history of head injury with loss of consciousness >10 minutes; ECT within the past 8 weeks; amblyopia (lazy eye) or a squint; diabetes; or hypertension  Groups were matched on age and parent education level | FEP and chronic SSD did not differ on any retinal vascular variable or CPZ  Combined SSD had reduced PD and VD, as well as enlarged FAZ size compared to controls  Nonsignificant negative correlation between illness duration and PD/VD, as well as a nonsignificant positive correlation with illness duration and FAZ size | Neurodevelopmental |
| Zhuo et al. (2021a) (18) | 97 FEP SZ with combined AH and VH  Divided FEP SZ into four groups:  1) n=20 with severe AH and VH (FUSCHSASV)  2) n=23 with middle-to-moderate AH combined with severe VH (FUSCHMASV)  3) n=28 with severe AH combined with middle-to-moderate VH (FUSCHSAMV)  4) n=26 with middle-to-moderate AH combined with middle-to-moderate VH (FUSCHMAMV)  30 controls | 22.0 (4.2)  26.4 (3.0)  25.2 (1.2)  27.9 (3.9)  25.4 (0.5) | Combined FEP SZ group: 0.35 (0.21) | SD-OCT (Cirrus HD-OCT 4000) | Excluded individuals with moderate to severe physical disease (e.g., respiratory, cardiovascular, endocrine, neurologic, liver, or kidney disease); current ECT treatment, a history of loss of consciousness >5 minutes for any cause; left-handedness; ophthalmic disease; high myopia; any MRI contraindication, including claustrophobia; IQ <80; any antipsychotic use for at least 3 weeks prior to the study; substance abuse; any other systemic disease, chronic disease, or head trauma, that can cause AH or VH; or retinal disease | Combined FEP SZ group had reduced total retinal thickness compared to controls, regardless of AH and VH severity | Neurodevelopmental |
| Zhuo et al. (2021b) (19) | 60 FEP SZ with visual disturbances  Divided FEP SZ into two groups:  1) n=30 with AH  2) n=30 without AH  30 controls | 23.7 (2.5)  24.5 (3.1)  24.0 (3.8) | Not reported | SD-OCT (Cirrus HD-OCT 4000) | Excluded individuals with moderate or severe liver disease; kidney disease; comorbidity of the respiratory, cardiovascular, endocrine, or nervous system; current ECT treatment; history of loss of consciousness for more than 5 minutes; left-handedness; severe myopia; ophthalmologic disease that can affect the retina; any MRI contraindication; antipsychotic medication taken ≥3 weeks prior to the study; IQ ≥80; or substance abuse  Groups were matched on age, gender, and education level | 85.0% of FEP SZ group had primary visual cortex retina co-impairments  Macular thickness was reduced in FEP SZ with AH compared to FEP SZ without AH and controls | Neurodevelopmental |
| Blose et al. (2024) (20) | 35,024 participants from the UK Biobank (40 SZ and 34,984 without SZ) | 56.8 (8.0) | N/A | SD-OCT (Topcon 3D-OCT 1000 Mark II) | Excluded individuals with non-European ancestry; missing PRS data; eye diseases; missing data on exposure variables; or OCT images that were of poor quality  Statistically controlled for sex, Townsend deprivation index, hypertension, diabetes, alcohol use, smoking status, cholesterol, and BMI | Higher polygenic risk for SZ was associated with reduced GCL-IPL thickness in individuals aged 40-49 and 50-59, but not in those aged 60-69  Greater polygenic risk for SZ was associated with reduced venular tortuosity, but not other microvascular indices | Neurodevelopmental |
| Boudriot et al. (2024) (21) | 103 SSD (SZ, SZA, and brief psychotic disorder; 42% of SSD in symptomatic remission)  130 controls | 39.08 (10.48)  33.58 (11.85) | 13.23 (8.75) | SD-OCT (Cirrus HD-OCT 5000) | Excluded individuals with concurrent clinically relevant central nervous system  diseases (MS and epilepsy); history of encephalitis, stroke, or cerebral  surgery; current pregnancy or breastfeeding; retinal pathologies due to a known eye condition or detected through OCT; increased IOP ≥21 mmHg, SPH $\leq$ −6 D or ≥ +6 D; or eyes with missing data on IOP and SPH  Statistically controlled for age, sex, SPH, IOP, BMI, diabetes, hypertension, and smoking status | SSD had reduced total macular, mRNFL, and IPL thickness compared to controls  Longer illness duration, more severe symptoms, higher CPZ, and poorer cognition was associated with altered ERG responses, changes in visual cortical areas, and reduced thickness of the IPL, RNFL, and HFL/ONL/MZ  Higher individual loading onto the disease-relevant signature of the visual system was associated with elevated polygenic risk for SZ | Neurodevelopmental and neurodegenerative |
| Fradkin et al. (2024) (22) | 28 SSD (SZ and SZA; n=12 FEP and n=16 chronic SSD)  37 controls (19 age-matched to FEP and 18 age-matched to chronic SSD) | 32.21 (11.27)  32.2 (12.63) | Not reported | SD-OCTA (Cirrus 5000 HD-OCTA) | Excluded individuals with eye injury or disease (e.g., cataracts, macular degeneration, diabetic retinopathy, glaucoma); neurological, intellectual, or developmental disorders; psychiatric diagnoses  of bipolar disorder or major depressive disorder (including past and current depressive episodes); a history of head injury with loss of consciousness greater than 10 minutes; ECT in the past 8 weeks; amblyopia (lazy eye) or squint; or self-reported or  chart diagnosis of diabetes or hypertension  Groups were matched on age and parental education | SZ had reduced PD, smaller vessel width, shorter vessel length, and reduced vessel network complexity in the deep vascular layer compared to controls  Chronic SSD had the lowest microvascular values, followed by FEP, followed by older controls (who did not differ from FEP), and followed by younger controls  Lower density values in the deep retinal layer were associated with lower density values in the superficial layer | Neurodevelopmental and neurodegenerative |
| Hosák et al. (2020) (23) | 39 SZ  39 healthy first-degree relatives of SZ participants (HR)  32 controls | 30.5 (7.1)  47.2 (11.5)  33.4 (9.1) | 9.7 (7.1) | SD-OCT (Cirrus HD-OCT) and digital fundus camera FF 450 + IR | Excluded individuals with hypertension, heart disease, stroke, or disorders potentially influencing retinal microvasculature, such as AMD; major deficits in visual acuity; or another known mental disorder  Statistically controlled for smoking status | SZ had increased CRAE compared to HR and controls (remained significant when restricting analyses to only non-smokers)  No significant differences in RNFL or GCL thickness between SZ and HR  SZ had reduced macula CSF thickness compared to HR  SZ had reduced nasal RNFL and average GCL-IPL thickness compared to controls  HR had increased CRAE, decreased inferior RNFL thickness, and GCL thickness (supranasal and supratemporal) compared to controls | Neurodevelopmental and neurodegenerative |
| Hosák et al. (2023) (24) | 53 SZ  53 healthy first-degree relatives of SZ participants (HR)  49 controls | 32.1 (9.6)  47.3 (12.7)  32.2 (8.4) | 10.4 (8.8) | Digital fundus camera (Zeiss FF450 + IR) | Excluded individuals with hypertension; heart disease; stroke; or disorders affecting retinal microvasculature (e.g., AMD or significant visual acuity deficits)  Statistically controlled for smoking status and BMI | Arteriolar and venular retinal diameters were widest in SZ, followed by HR, followed by controls—only significant difference was that SZ had increased retinal arteriolar diameters compared to controls (but not compared to HR)  Illness duration was not significantly correlated with retinal arteriolar or venular diameters | Neurodevelopmental and neurodegenerative |
| Krukow et al. (2024) (25) | 56 SZ  60 controls | 39.52 (15.38)  42.52 (10.9) | 14.83 (12.72)  Range: 0.25-42.0 | SD-OCT (Optopol Revo SD-OCT) | Excluded individuals with serious neurological or medical conditions; substance use disorder (except for nicotine); <12 years of education; ophthalmological conditions (e.g., glaucoma, macular degeneration, diabetic retinopathy,), trauma, or surgery; refraction higher  than ± 5 D; non-treated arterial hypertension or diabetes mellitus  Statistically controlled for BMI and smoking status  Groups were matched on age, education level, sex, and BMI | SZ had reduced macular and pRNFL thickness, macular volume, and GCC  Reduced macular thickness, macular volume, and GCC thickness were associated with longer illness duration  Greater severity of neurological soft signs was associated with reduced macular thickness, macular volume, and GCC thickness in SZ (but not in controls) | Neurodevelopmental and neurodegenerative |
| Liu et al. (2020a) (26) | 165 SZ  SZ was divided into 3 subgroups based on illness duration:  1) n=15 acute: <2 years  2) n=39 chronic: 2-10 years  3) n=112 long-term chronic: >10 years  72 controls | Whole SZ sample: 42.3 (11.49)  40.6 (8.3) | Whole SZ sample: 18.38 (11.44) | SD-OCT (Topcon-2001) | Excluded individuals with a history of hypertension, diabetes, eye or head trauma; history of drug abuse/addiction; glaucoma (IOP >21 mmHg); BCVA < 0.5, cataracts; high astigmatism, or congenital diseases of the optic nerve  Statistically controlled for age, sex, BMI, MAP, olanzapine equivalent dose, glucose levels, and cholesterol levels  Groups were matched on age, sex, smoking status, and BMI | SZ had greater mean retinal and vein diameters compared to controls, regardless of illness duration  SZ with family history of SZ had greater mean retinal vein diameters compared to SZ without a family history of SZ | Neurodevelopmental and neurodegenerative |
| Rabe et al. (2025) (27) | 34,939 participants from the UK Biobank | 56.87 (7.99) | N/A | SD-OCT (Topcon 3D-OCT 1000 Mark II) | Excluded individuals with antipsychotic use; eye diseases/disorders (diabetes-related eye diseases, glaucoma, macular degeneration, and injury/trauma resulting in vision loss); an ICD-10 diagnosis (F20-F29); highly myopic and hyperopic eyes (SPH ≥-6 D or SPH$\leq$ 3 D); incomplete data on BMI, smoking status, Townsend deprivation index, or alcohol use; with unmet SNP-level or sample-level quality control; or poor OCT image quality  Statistically controlled for age, sex, hypertension, diabetes mellitus, alcohol use, BMI, smoking status, Townsend deprivation index, OCT image quality, type of genotyping array used, and the first tend genetic principal components | Greater polygenic risk for SZ was associated with reduced inner and outer retina thickness  Greater polygenic risk score specific to neuroinflammation in relation to SZ were associated with reduced GCL-IPL thickness  No significant association found between retinal microvasculature gene-based polygenic risk scores for SZ and retinal phenotypes | Neurodevelopmental and neurodegenerative |
| Zhuo et al. (2020a) (28) | 48 FEP SZ with visual disturbance  50 controls | Baseline: 21.5 (1.7)  3-year follow-up: 24.5 (1.7)  Baseline: 22.0 (1.7)  3-year follow-up: 25.0 (2.3) | Not reported | SD-OCT (Cirrus HD-OCT 4000) | Excluded participants with antipsychotic use within 3 weeks prior to enrollment; contraindications for MRI; ophthalmologic diseases aﬀecting the retina; substance abuse; any disease that could cause visual disturbances (such as hyperglycemia, hyperlipidemia, or muscae volitantes); other systemic  diseases (e.g. respiratory, cardiovascular, endocrine, neurological, liver, or kidney disease); chronic diseases; history of loss of consciousness >5 minutes for any reason; previous head trauma; or IQ < 80  Groups were matched on age, sex, and education level | Total retinal thickness was reduced in FEP SZ compared to controls at baseline  More severe visual disturbance symptoms were reported in FEP SZ patients at 3-year follow-up  Total retinal thickness was reduced in FEP SZ after 3 years of antipsychotic treatment; no reduction in retinal thickness was observed among controls at 3-year follow-up  FEP SZ had reduced GMV in the visual cortex compared to controls and at 3-year follow-up, which correlated with retinal thinning  Reduction in GMV volume in the visual cortex and retinal thinning did not continue at 3.5-year follow-up | Neurodevelopmental and Neurodegenerative |
| Zhuo et al. (2020b) (29) | 97 FEP SZ with VH and AH  Divided FEP SZ into four groups:  1) n=20 with severe AH and VH (FUSCHSASV)  2) n=23 with middle-to-moderate AH combined with severe VH (FUSCHMASV)  3) n=28 with severe AH combined with middle-to-moderate VH (FUSCHSAMV)  4) n=26 with middle-to-moderate AH combined with middle-to-moderate VH (FUSCHMAMV)  30 controls | 22.0 (4.2)  26.4 (3.0)  25.2 (1.2)  27.9 (3.9  25.4 (0.5) | 1) FUSCHSASV: 0.2 (0.15)  2) FUSCHMASV: 0.27 (0.17)  3) FUSCHSAMV: 0.36 (0.13)  4) FUSCHMAMV: 0.52 (0.23) | SD-OCT (Cirrus HD-OCT 4000) | Excluded participants with any antipsychotic use for at least 3 weeks prior to the study; moderate-to-severe physical disease (e.g., respiratory, cardiovascular, endocrine, neurological, liver, or kidney disease); systemic, chronic, or any other disease that may cause AH or VH; head trauma; current ECT treatment; history of a loss of consciousness >5 minutes from any cause; left-handedness, ophthalmologic disease that can affect the retina; substance abuse; high myopia; any MRI contraindication (including claustrophobia); or IQ<80 | Combined FEP SZ sample had reduced retinal thickness compared to controls  No group differences found in retinal thickness across four FEP SZ groups  Total retinal thickness reduced in FEP SZ at 6-month follow-up—nasal parafoveal region had the largest reduction  No correlation found between retinal thickness reduction rate and CPZ | Neurodevelopmental and neurodegenerative |
| Alizadeh et al. (2021) (30) | 30 SSD males  Divided groups based on illness duration:  1) n=15 in an acute phase of illness  2) n=15 in a chronic phase of illness  15 male controls | 37.66 (9.24)  38.26 (7.9)  36.4 (9.51) | Total SSD sample: 13.82 (108.7)  Acute phase group: 12.20 (SD not reported)  Chronic phase group: 15.33 (SD not reported) | SD-OCT (Cirrus HD-OCT 5000) | Excluded individuals with other psychiatric issues (including substance use disorder); severe ophthalmologic conditions (e.g., glaucoma, AMD); media opacity with poor quality index in OCT BMI <18 or >28.8; refractive error >4 SPH D; severe metabolic/endocrinological issues; particularly high or low cortisol, cholestenone, or insulin concentrations; suicidal ideation; or unexpected general worsening of physical health  Statistically controlled for tobacco use  Groups were matched on age and gender | Longer illness duration was associated with increased pRNFL thickness in the acute phase group, while longer illness duration was associated with reduced pRNFL thickness in the chronic group  Acute phase group had reduced macula, pRNFL, and macula CSF thickness compared to controls  SSD participants with exclusively positive symptoms or exclusively negative symptoms had reduced macular thickness compared to SSD participants with a combination of positive and negative symptoms | Neurodegenerative |
| Antaki et al. (2025) (31) | 214 SZ  98,415 controls  Participants were from the AlzEye Cohort | 61.64 (12.05)  64.5 (13.5) | N/A | SD-OCT (Topcon 3D-OCT1000 and 3D-OCT2000) and SS-OCT (Topcon Triton Plus) | Excluded individuals with ICD-10 codes for all-cause dementia (E12, F00, F01, F02, F03, F106, F107, G30, G310); bipolar affective disorder (F30-F31); SSDs (excluding SZ, F21-F29); unipolar depression (F32-F33); unknown sex; images with non-macular fixation; or poor-quality images  Statistically controlled for age, sex, ethnicity, hypertension, diabetes, and SES | SZ was associated with a greater RAG compared to controls, but this lost significance after adjustment for diabetes and hypertension | Neurodegenerative |
| Appaji et al. (2019a) (32) | 98 SZ  87 BD  92 controls | 32.7 (6.0)  32.9 (6.0)  30.2 (7.8) | 7.6 (5.1) | 3nethra Classic (Non-mydriatic fundus camera with a 40-degree field view by Forus Health Pvt Itd) | Excluded individuals with substance use disorder (except  nicotine); comorbid psychiatric disorders; medical or neurological illness (hypertension, diabetes, cerebrovascular accident); or  history of ocular trauma  Statistically controlled for age, sex, nicotine use, BMI, and systolic blood pressure | Both SZ and BD had narrower retinal arterioles and wider venules compared to controls  BD had narrower arterioles and wider venules compared to SZ  No significant association found between retinal vascular diameters and clinical variables (illness duration, number of episodes, or severity of symptoms) | Neurodegenerative |
| Appaji et al. (2019b) (33) | 94 SZ  88 BD  87 controls | 32.5 (6.0)  33.4 (6.0)  30.2 (7.6) | 7.6 (5.2) | 3nethra Classic (Non-mydriatic fundus camera with a 40-degree field view by Forus Health Pvt Itd) | Excluded individuals with an  ongoing psychiatric comorbidity; substance abuse or dependence (except nicotine) in the previous 12 months; hypertension; diabetes; stroke; history of eye surgery/trauma; or ophthalmologic disorders (e.g., macular degeneration, glaucoma)  Statistically controlled for age, sex, nicotine use, systolic blood pressure, and BMI | SZ and BD had wider and flatter parabola of retinal arteries and steeper and narrower parabola of retinal veins compared to controls  No significant correlations found between retinal vascular trajectories and the clinical variables (illness duration, age at onset of illness, symptom severity, and CPZ) | Neurodegenerative |
| Appaji et al. (2019c) (34) | 79 SZ  86 BD  78 controls | 32.7 (6.0)  33.2 (6.2)  30.0 (7.6) | 7.7 (5.1) | 3nethra Classic (Non-mydriatic fundus camera with a 40-degree field view by Forus Health Pvt Itd) | Excluded individuals with concurrent substance abuse or dependence (except nicotine); comorbid Axis I psychiatric disorders; serious medical conditions (e.g., hypertension, diabetes, stroke); or history of eye trauma and surgery  Statistically controlled for age, sex, nicotine use, systolic blood pressure, and BMI | SZ and BD had greater RATI (but not RVTI) compared to controls  No significant correlations found between RATI and clinical variables (symptom severity, age at onset of illness, and illness duration) | Neurodegenerative |
| Ascaso et al. (2015) (35) | 30 SZ (n=10 with recent illness episode and n=20 with nonrecent illness episode)  30 controls | 45.1 (10.4)  44.5 (10.9) | 16.3 (11.2) | TD-OCT (Stratus OCT) | Excluded individuals with a history of or current systemic disease that may affect the eyes; history of ophthalmological or neurological disease known to affect the visual pathway; media opacification (e.g., such as cataract, corneal leukoma, or vitreous hemorrhage) that could impede ocular and OCT examination; refractive error ≥± 2 SPH D; a history of head injury with loss of consciousness; or drug/alcohol dependence  Groups were matched on age and gender | pRNFL, foveal, and macular thickness, as well as macular volume were reduced in combined SZ group compared to controls  No significant correlation found between retinal indices and illness duration  Nonrecent illness episode SZ had reduced pRNFL thickness, macular thickness, and macular volume compared to controls | Neurodegenerative |
| Bannai et al. (2020) (36) | 25 PSD (n=12 SZ, n=8 SZA, and n=5 BD with psychotic features)  15 controls | 36.2 (13.2)  39.0 (12.6) | 11.88 (13.47) | SD-OCT (Spectralis OCT) | Excluded individuals with a history of substance  dependence or abuse within the past 6 months; ophthalmological conditions (e.g., glaucoma, macular  degeneration, retinal occlusions, ocular trauma or myopia >4.0 D); current pregnancy/breast feeding; head injury with neurological intellectual disability; or a history of neurologic disorders  Statistically controlled for age, sex, race, BMI, systolic and diastolic BP, mean visual acuity, cardiometabolic disorder, smoking status, and antipsychotic status  Groups were matched on age, sex, race, visual acuity, BMI, cardiometabolic disease status, and smoking status | PSD had reduced ONL thickness and increased OPL thickness compared to controls  Reduced RPE thickness was associated with more severe mania symptoms  Reduced ONL thickness was associated with poorer overall cognitive functioning  Reduced ONL and total retina thicknesses were associated with smaller total brain volume; reduced ONL thickness was associated with smaller white matter volume | Neurodegenerative |
| Blose et al. (2023) (37) | 60 SSD (SZ and SZA) (n=11 with diabetes or hypertension)  69 controls (n=11 with diabetes or hypertension) | 36.67 (12.35)  35.45 (12.34) | 10.76 (10.59) | SD-OCT (Cirrus HD- OCT 4000 or 5000) | Excluded individuals with retinal or other eye injuries or disease (e.g., macular degeneration, diabetic retinopathy, glaucoma); neurological disorders; intellectual disorders; developmental disorders; or a head injury resulting in a loss of consciousness >10 minutes  Groups were matched on age, sex, and hypertension/diabetes status | Age was negatively correlated with pRNFL thickness, GCL-IPL thickness, and macula volume was reduction in SSD, but not in controls; the age relationship with pRNFL thickness and macula volume in SSD lost significance when participants with diabetes/hypertension were removed, but the age relationship with GCL-IPL thickness remained significant  Longer illness duration was associated with reduced pRNFL and GCL-IPL thickness, as well as reduced macula volume | Neurodegenerative |
| Boudriot et al. (2023) (38) | 65 SSD (47 SZ, 17 SZA, 1 BD)  89 controls | 39.29 (10.8)  34.65 (11.35) | 13.69 (7.81) | SD-OCT/OCTA (Cirrus HD- OCT 5000) | Excluded individuals with a concurrent clinically relevant central nervous system disorder; a history of encephalitis, meningitis, or stroke; retinal pathology; IOP ≥21 mmHg; refractive error ≥±6 SPH D; or pregnancy  Statistically controlled for age, sex, SPH, IOP, BMI, diabetes, hypertension, smoking status, and OCT signal strength  Groups were matched on sex, hypertension status, and BCVA | Longer illness duration and higher CPZ were associated with reduced parafoveal macular and mRNFL thickness  SSD group had significantly reduced GCL-IPL, mRNFL, and pRNFL thickness compared to controls  No significant group differences in PD, VD, or FAZ size | Neurodegenerative |
| Budakoglu et al. (2021) (39) | 22 SZ  26 controls | 40.8 (7.9)  45.2 (8.1) | 8.3 (3.3) | SD-OCT (Optovue RTVue XR Avanti OCT) | Excluded individuals with any pathology in the anterior segment  and/or posterior segment examination; a history of ocular surgery; corrected IOP >21 mm Hg; refractive error ≥±1 SPH/CYL D; systemic diseases affecting the retinal vascular structures (e.g., diabetes, hypertension); media opacity affecting the imaging; illness duration $\leq$2 years; or age <18 years  Groups were matched on age, gender, and IOP | SZ had reduced pRNFL thickness compared to controls  SZ had lower vascular density compared to controls  Longer illness duration was associated with greater RPC VD, but this did not reach significance | Neurodegenerative |
| Celik et al. (2016) (40) | 40 treatment-resistant SZ  41 treatment-responsive SZ  41 healthy controls | 35.75 (10.7)  35.37 (9.69)  35.49 (16.0) | Total SZ sample: 13.28 (9.15)  Treatment-resistant: 14.55 (8.28)  Treatment-responsive: 12.04 (9.87) | SD-OCT (Spectralis OCT) | Excluded individuals with a(n) degenerative neurologic, immunologic, or systemic illness; ophthalmologic condition; or refractive error >±1 SPH/CYL D  Groups were matched on age and sex | Longer illness duration and greater number of hospitalizations were associated with reduced choroid thickness in the combined SZ sample  pRNFL thickness, as well as GCL and IPL volumes were significantly reduced in SZ group compared to control group  GCL and IPL volumes were significantly reduced in treatment-resistant group compared to treatment-responsive group  Age was negatively correlated with right choroid, GCL, and IPL thickness (but not pRNFL)  PANSS score was negatively associated with right choroid and GCL (but not IPL or pRNFL) thickness  More severe psychotic illness was correlated with reduced GCL and IPL thickness | Neurodegenerative |
| Delıbaş et al. (2018) (41) | 63 SZ (most of whom were chronically ill) divided into two groups based on degree of insight into illness:  1) n=31 with insight  2) n=32 without insight  39 controls | 42.13 (9.82)  39.84 (11.82)  40.13 (9.03) | 1) M=with insight: 15.84 (8.81) 14.64 years (SD=9.9 years)  2) without insight: 13.47 (10.86) | SD-OCT (Cirrus HD-OCT 4000) | Excluded individuals with medical conditions that may affect the CNS or retina (e.g., diabetes mellitus); alcohol and/or substance abuse in the past 6 months; previous ocular surgery or trauma; history of ocular diseases (such as mature cataract, corneal opacity, uveitis, vitreous hemorrhage, glaucoma, retinal vascular disease); intellectual disability; or significant symptom exacerbation in the last 6 months  Groups were matched on age, gender, and education level | Illness duration was not correlated with any retinal indices  Combined SZ group had reduced pRNFL and GCL-IPL thickness compared to controls  SZ patients without insight had reduced pRNFL and GCL-IPL thickness compared to SZ patients with insight and controls | Neurodegenerative |
| Domagała et al. (2023) (42) | 60 SZ divided into 3 age groups:  1) n=20 (Y; 20-31 years)  2) n=21 (M; 32-45 years)  3 n=19 (O; 46-65 years)  61 controls divided into same 3 age groups:  1) n=19 Y  2) n=23 M  3) n=19 O | 25.5 (3.67)  38.35 (6.24)  60.11 (5.24)  28.0 (3.91)  42.5 (3.36)  57.22 (2.42) | Y: 5.53 (3.81)  M: 15.91 (8.33)  O: 31.47 (11.74) | SD-OCT (Optopol Copernicus REVO) | Excluded individuals with serious neurological conditions; serious medical conditions; a history of psychoactive substance (excluding nicotine) addiction in the past 6 months; any previously diagnosed ophthalmological disorders (e.g., glaucoma, macular degeneration, and diabetic retinopathy); diabetes mellitus, non-treated arterial hypertension (if treated, BP should be > 140/90 mm Hg during an evaluation); obesity (BMI > 30); history of ocular trauma or surgery; eye refraction > ± 5 D; glaucoma suspect (DDLS≥ 6); dementia; or any relevant, concomitant psychiatric disorder (F25.x)  Controlled for risperidone equivalent dose  Groups were matched on age, gender, and level of education | Macular thickness and volume showed an age-related decrease in SZ but not it controls. This decrease occurred primarily between 32 and 45 years of age | Neurodegenerative |
| Hanifi Kokaçya & İdil Çakmak (2022) (43) | 47 SZ divided into two groups based on illness duration:  1) n=23 short-term SZ (illness duration ≤5 years)  2) n=24 long-term SZ (illness duration >5 years)  50 controls | 34.74 (10.86)  30.3 (8.98)  39.0 (10.6)  34.02 (10.3) | N/A | SD-OCT/OCTA (Optovue RTVue-XR Avanti OCT) | Excluded individuals with visual acuity <20/25 and refractive error ≥2 D; a history of pregnancy; substance use; systemic disease; or ophthalmologic conditions  Combined SZ and controls were matched on age and gender | Combined SZ sample had significantly reduced pRNFL thickness, macula thickness, and VD compared to controls  Long-term patients had significantly reduced VD of RCP, SCP, and RPC compared to short-term patients  More severe illness was associated with increased perifoveal macular thickness and VD of RCP  Longer illness duration was associated with decreased VD of the SCP, DCP, and reduced perifovea thickness | Neurodegenerative |
| Jerotic et al. (2020) (44) | 33 “non-acute” PSD (SZ, SZA, and psychosis unspecified)  35 controls | 33.1 (6.1)  32.5 (9.9) | 6.5 (4.3) | SD-OCT (Cirrus HD-OCT 4000) | Excluded individuals with hypertension, diabetes, or other systemic/neurologic disorders known to affect the visual system; refractive error ≥±2 SPH D; any ophthalmologic condition; previous head injury with loss of consciousness; history of substance or alcohol abuse; or intellectual disability  Groups were matched on age, sex, and education level | PSD had significantly reduced GCL-IPL, macula average, and macula CSF thickness, as well as reduced macula volume  No significant group differences in pRNFL thickness  Longer illness duration was associated with significantly reduced pRNFL thickness | Neurodegenerative |
| Jerotic et al. (2021) (45) | 42 “non-acute” PSD (SZ, SZA, and psychosis unspecified)  35 controls | PSD males: 33.8 (6.5)  PSD females: 31.2 (7.3)  Control males: 30.0 (6.4)  Control females: 34.5 (11.5) | Total PSD sample: 6.06 (4.39)  PSD females: 4.8 (3.6)  PSD males: 7.2 (4.8) | SD-OCT (Cirrus HD-OCT 4000) | Excluded individuals with hypertension, diabetes, or other systemic/neurologic disorders known to affect the visual system; refractive error ≥±2 SPH D; any ophthalmologic condition; previous head injury with loss of consciousness; history of substance or alcohol abuse; IQ$\leq$80; or illness duration >15 years | PSD had reduced macular and GCL-IPL thicknesses compared to controls  PSD females had thinner macular and GCL-IPL thicknesses than males | Neurodegenerative |
| Joe et al. (2018) (46) | 6 patients with psychosis (n=3 SZ and n=3 BD with psychotic features)  18 controls | 56.0 (8.9)  56.5 (8.0) | Range: 16-39 | SD-OCT (Spectralis HRA+OCT) | Excluded individuals with any preexisting retinal and ocular pathology (including macular degeneration, glaucoma, and diabetic retinopathy) | Patients had reduced macular thickness compared to controls  No significant association was found between retinal indices and illness duration | Neurodegenerative |
| Kango et al. (2023) (47) | 35 SZ in remission (SZCR)  35 treatment-resistant SZ (TRS)  21 FEP SZ (antipsychotic-naïve)  36 controls | 31.17 (7.73)  29.85 (7.01)  30.33 (6.47)  31.0 (3.68) | Combined SZ sample: 6.05 (5.63)  SZCR: 6.22 (4.68)  TRS: 8.67 (6.31)  FEP: 1.40 (1.78) | SS-OCT (DRI Topcon Triton) | Excluded individuals with an obscured clarity of media due to the presence of cataract, vitreous haze, or any other such coexistent pathology that did not allow the acquisition of good images; refractive errors ≥±3 D; ophthalmologic condition; congenital retinal pathologies; Parkinson’s disease; organic brain syndrome; intellectual disability; diabetes mellitus; hypertension; dementia, MS; HIV; head injury; epilepsy; encephalopathy; co-morbid psychiatric disorders (other than a lifetime diagnosis of depression, obsessive–compulsive disorder, tobacco dependence syndrome); current major depressive disorder; or current obsessive-compulsive disorder  Statistically controlled for age, gender, duration of treatment, smoking status, current medications, BMI, waist circumference, blood pressure, fasting glucose, HbA1c, and the presence or absence of metabolic syndrome | SZCR group had significantly reduced sub-foveal retinal thickness and macular volume compared to controls  No significant differences in retinal indices between TRS group and controls after adjustments for covariates  No significant differences in retinal indices between FEP and controls after adjustments for covariates  SZCR had reduced left ganglion cell thickness compared to TRS  SZCR had reduced left RNFL thickness compared to FEP  TRS had reduced sub-foveal choroidal thickness and left central 1 mm thickness of retina compared to FEP | Neurodegenerative |
| Khalil et al. (2022) (48) | 30 SZ males (included chronic patients and those having an “acute attack”)  30 controls | 30.16 (5.6)  29.73 (5.18) | 7.35 (4.5) | SD-OCT (Optovue RTVue OCT) | Excluded individuals with diseases that may affect OCT parameters (e.g., diabetes mellitus, hypertension); myopia >2.0 D; glaucoma; optic neuropathies; macular degeneration; eyes with media opacities that may preclude OCT measurement or interfere with ocular examination; previous hypotensive crisis; ocular surgery within <3 months; or intraorbital or intracranial lesions that may interrupt the visual pathway  Groups were matched on age, BCVA, slit-lamp exam findings, and fundus exam findings | pRNFL thickness was reduced in SZ compared to controls  No group differences found for GCC thickness  Longer illness duration was associated with reduced pRNFL thickness | Neurodegenerative |
| Kozub et al. (2020) (49) | 12 SSD (n=11 paranoid SZ and n=1 with SPD)  Further divided patients based on disease duration: 1) Subgroup A (n=6): Disease duration > 5 years; 2) Subgroup B (n=6): Disease duration < 5 years  10 out 12 patients were inpatients with “acute disease” and 2 were in remission for more than a year during the study  12 controls | 27.6 (range: 18-37)  28.3 (range: 19-36) | Combined SSD group: 8.5 (5.4) | SD-OCT (Topcon 3D OCT 2000) | Excluded individuals with high myopia with secondary changes in the fundus or ophthalmological condition  Groups were matched on age and gender | SZ had significantly increased macula thickness and volume  Subgroup A (disease duration > 5 years) had significantly increased macular volume and thickness compared to controls  Subgroup B (disease duration < 5 years) had no significant differences in macular parameters compared to controls | Neurodegenerative |
| Krukow et al. (2025) (50) | 124 SSD (n=59 SZ from a Polish database and n=65 SZ from a US database)  130 controls (n=60 controls from PL database and n=70 from US database) | 38.39 (12.24)  39.29 (10.06) | Not reported | SD-OCT (Optopol Revo SD-OCT) and (Cirrus HD-OCT 4000 and 5000) | Poland sample: Excluded individuals with current or a history of ophthalmological conditions (e.g., glaucoma, diabetic retinopathy, macular degeneration); ophthalmological surgery or injury; diabetes; intellectual impairment; traumatic brain injury; neurovascular lesions; or substance use disorder  US sample: Excluded individuals with eye disease or injury; neurologic illness; neurodevelopmental disorder; intellectual impairment; or head injury with loss of consciousness > 10 minutes  Statistically controlled for hypertension, BMI, and smoking | Retinal age gap was higher in SZ than in controls  Retinal age gap was higher in younger than in older SZ patients  Higher retinal age gap was associated with more severe negative and general psychopathology symptoms, as well as with greater CPZ | Neurodegenerative |
| Kurt et al. (2022) (51) | 44 SSD (n=37 SZ and n=7 SZA)  41 controls | 47.82 (9.44)  45.59 (9.92) | 24.79 (15.22) | SD-OCT (Cirrus HD-OCT) | Excluded individuals with a history of ophthalmological conditions that could affect OCT (e.g., diabetic or hypertensive retinopathy); refractive error >± 3.0 SPH D and/or >± 2.0 CYL D, IOP >21 mm Hg; low-quality OCT scans (signal strength ≤6); intellectual disability; comorbid psychiatric disorders; or neurodegenerative diseases (AD, Parkinson’s disease, or MS)  Groups were matched on age, gender, systemic disease status, and IOP | All choroid and mRNFL thicknesses were significantly reduced in SSD compared to controls  Longer illness duration was associated with reduced nasal choroidal diameter (but not for other retinal measures)  Positive correlation between PANSS total scores and choroid thickness  Positive correlation between mRNFL thickness and PANSS negative symptom scores | Neurodegenerative |
| Kurtulmus et al. (2023) (52) | 43 chronic SZ  40 controls | 42.38 (10.95)  38.9 (11.91) | 18.44 (9.84) | SD-OCT (Spectralis OCT) | Excluded individuals with any ophthalmologic, neurologic, or systemic condition/disease known to affect the visual system; previous or concurrent autoimmune diseases; SPH of refractive error of <−6 D or >+6 D; intellectual disability; illiteracy; acute psychosis; comorbid Axis 1 psychiatric disorder; or substance or alcohol misuse  Statistically controlled for comorbid medical diseases, BMI, and tobacco smoking, as well as glucose, HDL, and triglyceride levels  Groups were matched on gender, age, smoking status, HDL, triglycerides, and CRP | SZ had reduced IPL compared to controls  No significant group differences found for RNFL, GCL, or choroid thickness  Higher IL-6, IL-1β, and TNF-α levels were associated with reduced macular thickness and higher IL-6 was associated with reduced IPL and choroid thickness in the overall sample  Reduced IPL and macula thickness were associated with worse executive functioning and attention  In SZ, reduced IPL thickness was associated with increased BMI and decreased HDL levels  Decreased TNF-α level was associated with reduced IPL thickness in SZ | Neurodegenerative^[[4]](#footnote-4)^ |
| Lai et al. (2020) (53) | 33 SSD (SZ and SZA) divided into two subgroups based on illness duration:  1) n=15 FEP  2) n=18 chronic  38 controls divided into two subgroups:  1) n=20 <30 years old  2) n=18 >30 years old | 24.6 (6.45)  39.67 (11.03)  23.1 (2.29)  41.89 (11.79) | 17.76 (21.50) | SD-OCT (Cirrus HD-OCT 5000) | Excluded individuals with a history of ophthalmologic condition/disease; history of head injury with loss of consciousness of more than 10 minutes; ECT within the past 8 weeks; amblyopia (lazy eye) or a squint; diabetes, high blood pressure; neurologic, intellectual, mood, or developmental disorder | Combined SSD group had significantly thinner macula CSF compared to controls; no significant differences between the groups for pRNFL thickness, CDR, macula volume, or GCL-IPL thicknesses  FEP did not have significant differences on any of the retinal indices between age-matched controls  Chronic SSD had significantly thinner macula CSF values and decreased macula volume compared to their age-matched controls; no significant differences in pRNFL thickness, CDR, or GCL-IPL thicknesses  Chronic SSD had significantly thinner macula CSF and GCL-IPL compared to FEP | Neurodegenerative |
| Lee et al. (2013) (54) | 30 SZ divided into subgroups according to illness duration:  1) Acute (n=5)  2) Chronic (n=13)  3) Long-term chronic (n=12)  30 controls | 37.17 (10.67)  35.97 (9.1) | 1) Acute: illness duration $\leq$2 years  2) Chronic: illness duration >2 years and $\leq$10 years  3) Chronic: illness duration >10 years | SD-OCT (Cirrus HD-OCT 4000) | Excluded individuals with ophthalmologic conditions/diseases that might affect the retinal nerve fiber structures; previously known history of hypotensive crisis; history of intracranial or intraorbital space-occupying lesions that can affect the visual pathway; diabetes mellitus; refractive error >−2.0 SPH D; or significant media opacities precluding ocular examination or OCT measurement  Groups were matched on age, sex, and ethnicity | pRNFL and macula thickness, as well as macula volume, were reduced in combined SZ sample compared to controls  Longer illness duration was associated with reduced pRNFL and macular thickness, as well as reduced macula volume  Both chronic SZ groups had reduced pRNFL thickness compared to controls and acute SZ  Macula thickness and volume were reduced in both chronic SZ groups compared to controls  Macula thickness and volume were reduced in chronic SZ (illness duration >10 years) compared to acute SZ  No differences in macula thickness and volume between both chronic SZ groups nor between acute SZ and controls | Neurodegenerative |
| Liu et al. (2020b) (55) | 221 SZ  149 controls | 44.2 (12.02)  41.59 (9.37) | 20.11 (12.45) | SD-OCT (Topcon 2002 type SD-OCT) | Excluded individuals with acute and chronic medical conditions, including inflammation, pregnancy, hypertension, head trauma, diabetes mellitus, drug and alcohol abuse or addiction, glaucoma, cataracts, high astigmatism (sphere D >4 and cylinder D >2); or optic media opacities  Statistically controlled for smoking status, BMI, mean arterial pressure, serum glucose, cholesterol, uric acid, alanine aminotransferase, and family history of SZ | pRNFL and macular thicknesses were reduced in SZ compared to controls  Macular thickness did not significantly correlate with serum CNTF or illness duration  Longer illness duration was associated with reduced serum CNTF and reduced pRNFL thickness  Reduced pRNFL thickness was associated with poorer cognitive functioning | Neurodegenerative |
| Liu et al. (2021) (56) | 138 SZ  160 controls | 45.09 (12.47)  41.43 (9.5) | 21.74 (13.51) | SD-OCT (Topcon 2002 type SD-OCT) | Excluded individuals with central nervous system diseases; head trauma; hypertension; diabetes mellitus; drug/alcohol use disorder; glaucoma; cataracts; high astigmatism (>4 SPH D and >2 CYL D); high myopia (>6.0 D); visual field defect; optic media opacities; chromatodysopia; or any other ophthalmologic conditions/diseases; as well as other significant medical illnesses (e.g., cancer, ongoing infection)  Statistically controlled for age, sex, and smoking status  Groups were matched on BMI and years of education | SZ had reduced pRNFL thickness compared to controls  Longer illness duration was associated with reduced pRNFL thickness  Decreased VEGF was associated with reduced pRNFL thickness in SZ | Neurodegenerative |
| Miller et al. (2020) (57) | 12 SSD (n=8 SZ, n=3 SZA, and n=1 DD)  12 controls | 48.25 (10.29)  48.29 (10.64) | 27.25 (14.41) | SD-OCT (Spectralis OCT, Cirrus 4000 OCT) | Excluded individuals with a history of neurologic disorders or ophthalmologic diseases | SZ had reduced macular volume on both Spectralis and Cirrus devices compared to controls  No group differences were found for pRNFL or GCL-IPL | Neurodegenerative |
| Mota et al. (2015) (58) | 20 SZ divided into 2 subgroups:  1) n=10 FEP SZ  2) n=10 chronic SZ  20 controls | 32.9 (11.9)  23.8 (9.9)  42.0 (3.9)  33.4 (11.2) | 1) FEP SZ <5 years  2) Chronic SZ >5 years | SD-OCT (Spectralis OCT) | Excluded individuals with degenerative or neurologic diseases; general systemic diseases that could affect the eye (e.g., autoimmune or infectious diseases); history of traumatic brain injury associated with loss of consciousness; episodes of acute decompensation; current history of prominent addictive behavior (alcohol or other illegal drugs); history of macrostructural lesions of the central nervous system; other relevant comorbid psychiatric disorders (particularly, intellectual disability or dementia); refractive error ≥−6.00 D, BCVA <8/10, IOP ≥22 mm Hg; opacification of the transparent media of the eye; change in excavation/upper disc >0.4; or pathology of the retinal posterior pole (diabetic retinopathy, hypertension, etc.) | Reduced macular thickness and volume in combined SZ sample compared to controls  Reduced macular thickness and volume in chronic SZ compared to FEP SZ  Longer illness duration was associated with reduced pRNFL thickness | Neurodegenerative |
| Murav’eva et al. (2021) (59) | 14 paranoid SZ  30 controls (age range: 18-35 years) | Range: 21-40 years  Range: 18-35 years | 6.7 (3.4) | SD-OCT (Topcon 3D OCT 2000) | Excluded individuals with extreme myopia, any macular pathology, or any neuro-opticopathy | Macula thickness and volume were increased in SZ compared to controls | Neurodegenerative |
| Orum et al. (2020) (60) | 82 SZ divided into 3 subgroups:  1) n=26 SZ on clozapine (CG)  2) n=34 SZ on other second-generation antipsychotics (SGAG)  3) n=22 SZ on first-generation antipsychotics (FGAG)  Patients were also divided into two other subgroups:  1) n=44 treatment-resistant SZ (TRS)  2) n=38 non-treatment resistant SZ (N-TRS)  50 controls | 36.76 (10.63)  33.0 (8.83)  36.67 (10.93)  41.36 (10.74)  41.02 (13.69) | Whole SZ sample: 14.48 (9.55)  CG: 12.73 (8.02)  SGAG: 12.58 (10.16)  FGAG: 17.95 (9.76)  TRS: 16.29 (9.01)  N-TRS: 12.36 (9.84) | SD-OCT (SpectralisTM OCT) | Excluded individuals with comorbid first axis diagnosis; hypertension; diabetes mellitus; severe neurological diseases; immunological diseases; drug use other than antipsychotics; or refraction errors ≥1 prism D  Groups were matched on age; SZ groups were matched on illness duration | pRNFL thickness was reduced in both TRS and N-TRS SZ groups, regardless of antipsychotic group status, compared to controls  No difference in choroid layer between N-TRS and controls  TRS had reduced choroid layer compared to controls  TRS had reduced choroid layer, GCL, and IPL thickness compared to N-TRS  No differences in pRNFL thickness between patients grouped by antipsychotic status  No differences found for GCL or IPL thickness between FGAG, CG, and SGAG  Choroid layer thickness was increased in SGAG compared to CG, FGAG, and controls | Neurodegenerative |
| Padmanabhan et al. (2024) (61) | 36 SZ in remission for at least 6 months | n=8: 20-30 years  n=10: 31-40 years  n=18: 41-50 years | Range: 5 to 30 years | SD-OCT (DRI OCT Triton plus-3D OCT) | Excluded individuals with a history of concurrent systemic disease that may aﬀect the eye; history of an ophthalmological or neurological disease known  to aﬀect the visual pathway; media opacification (e.g., cataract, corneal leukoma, or vitreous hemorrhage that may impede ocular or OCT examination); refractive errors over +2 SPH D or hyperopic defects or -2 SPH D for myopic defects; history of head injury with loss of consciousness; intellectual disability; or substance use | Poorer cognitive functioning was associated with reduced RNFL thickness  No significant correlation found between illness duration and RNFL thickness, macular thickness, or macular volume | Neurodegenerative |
| Samani et al. (2018) (62) | 35 SZ  50 controls | 40.6 (12.9)  40.6 (12.7) | 16.3 (9.1) | SD-OCT (Leica Envisu hand-held SD-OCT) | Excluded individuals with a history of any ophthalmologic diseases/conditions; diabetes mellitus; refractive error ≥±6 SPH D; or substance dependency  Statistically controlled for age, gender, ethnicity, and SPH  Groups were matched on age, gender, and ethnicity | Total retinal and photoreceptor complex thicknesses, as well as ISL and ONL and parafoveal ganglion cell region, were reduced in SZ compared to controls  Longer illness duration was associated with decreased temporal parafoveal ISL, temporal parafoveal ONL, and temporal parafoveal photoreceptor complex thicknesses  Low spatial frequency contrast sensitivity was reduced in SZ and correlated with reduced temporal parafoveal GCC thickness  Negative PANSS symptom severity was negatively correlated with foveal photoreceptor complex and ONL thickness | Neurodegenerative |
| Schönfeldt-Lecuona et al. (2020) (63) | 26 SSD (n=17 SZ and n=9 SZA)  23 controls | 37.0 (10.9)  40.3 (11.6) | 10.2 (10.2) | SD-OCT (Spectralis OCT) | Excluded individuals with ophthalmologic disorders or systemic diseases that could affect the optic nerve or the retina (such as diabetes, glaucoma, or refractory arterial hypertension); neurologic diseases affecting the brain; clinically relevant cognitive impairment revealed by an MMSE score of <26; current substance abuse; high-dose steroid therapy; recent eye surgery; autoimmune diseases affecting the eye; IOP >21 mm Hg; or refractive error >6 D  Groups were matched on age, gender, and BMI | Macular thickness, macular volume, INL volume, ONL thickness, and mRNFL thickness were reduced in SSD compared to controls  Longer illness duration was associated with reduced mRNFL volume | Neurodegenerative |
| Tasdelen et al. (2023) (64) | 36 SZ  36 healthy siblings of SZ participants (age: M=26.0 years, range: 21.25-37.5 years) | 29.5^[[5]](#footnote-5)^ (25.25-37.0^[[6]](#footnote-6)^  26.0^5^ (21.25-37.5)^6^ | 11.77 (7.38) | SD-OCT (Optopol Revo SD-OCT) | Excluded participants with ophthalmologic diseases; diseases affecting the retina (e.g., diabetes mellitus,  high blood pressure, epilepsy, or history of serious head injury); fasting glucose levels outside of normal range; history of intellectual disability; pervasive developmental disorder; active substance/alcohol use; or current ECT treatment  Statistically controlled for gender, age, and BMI  Groups were matched on age, gender, and smoking status | SZ had reduced GCL-IPL thickness (across various segments) and macular volume, compared to their siblings; pRNFL thickness was similar across the groups  Among SZ, no significant correlations found between OCT indices and symptom severity, level of global functioning, or overall severity of illness  Reduced pRNFL thickness was associated with poorer working memory  Reduced GCL-IPL thickness was associated with poorer verbal fluency | Neurodegenerative |
| Topcu-Yilmaz et al. (2019) (65) | 59 SZ (n=8 acute and n =51 chronic patients)  37 controls | 34.64 (9.49)  32.08 (12.33) | 10.33 (SD not reported) | SD-OCT (Spectralis OCT) | Excluded individuals with history of an ophthalmologic, neurologic, or systemic condition that could affect the RNFL or macula (such as amblyopia, glaucoma, age-related macular degeneration, diabetes mellitus, hypertension, optic neuropathy, ocular trauma, ocular surgery, IOP ≥21 mm Hg, etc.); refractive errors ≥± 2.0 SPH D; or media opacities that preclude OCT examination  Groups were matched on age and gender | Macular thickness was reduced in SZ compared to controls  No differences in pRNFL or choroid thickness between SZ group and controls  Longer illness duration was associated with reduced choroid thickness | Neurodegenerative |
| Wagner et al. (2023) (66) | 485 SZ  100,931 controls | 64.9 (12.2)  65.9 (13.7) | Not reported | SD-OCT, SS-OCT (Topcon Inc) | Excluded individuals with a mental illness other than SZ; unknown sex; poor image quality; or nonmacular fixation  Statistically controlled for age, sex, SES, diabetes, hypertension, and image quality  Groups were matched on age, sex, glaucoma status, and AMD status | SZ had thinner GCL-IPL and enlarged CDR compared to controls  Younger individuals with SZ (age <55 years) had a more modest reduction in GCL-IPL thickness compared to older individuals with SZ (age ≥55 years)  SZ had reduced retinal fractal dimension, increased tortuosity, and increased vascular caliber compared to controls; however, most of these differences were accounted for by diabetes or hypertension | Neurodegenerative |
| Zhu et al. (2024) | 29 SZ  25 controls | 39.93 (14.01)  39.23 (11.82) | 16.48 (12.62) | SD-OCT (Mocean 4000 OCT) | Excluded individuals with cataract, glaucoma, ocular injury or other severe ocular pathology; substance abuse; an inability to cooperate with examination; intellectual disability; comorbid diabetes, hypertension, neurological disease, neurotrauma history or any other medical condition that could lead to neural retina degenerative deficits; refractive error > ± 5.0 diopters; IOP <10 mmHg or >21 mmHg; ECT over last 4 weeks  Statistically controlled for age and sex  Groups were matched on age, sex, D, and IOP | SZ had reduced CFT, macular thickness, and GCC thickness compared to controls  SZ had larger optic cup volume compared to controls  More severe negative symptoms were associated with reduced total macular and CFT thickness  and  No significant associations found between any of the other OCT parameters and positive symptom severity, CPZ, or illness duration | Neurodegenerative |
| Altun et al. (2020) (67) | 35 SZ (who had been using risperidone or clozapine for at least 6 months)  36 BD  31 controls | 44.2 (10.02)  42.17 (13.39)  39.35 (4.27) | 20.3 (8.91) | SD-OCT (Optovue RTVue Premier OCT) | Excluded individuals with intellectual disability, additional medical, psychiatric, or neurologic disease; additional antipsychotic use at a dose exceeding CPZ of 300mg; ophthalmologic conditions; hypertension; diabetes; intracranial or intraocular lesions; or history of brain surgery  Groups were matched on age and gender | No significant difference in macular or pRNFL thickness between the three groups  In SZ group, CPZ of risperidone had a negative association with pRNFL thickness | Neither |
| Appaji et al. (2020) (68) | 34 SZ  34 BD  45 controls | 31.8 (5.0)  32.9 (6.0)  32.8 (8.0) | Not reported | 3nethra Classic (Non-mydriatic fundus camera with a 40-degree field view by Forus Health Pvt Itd) | Excluded individuals with substance abuse or dependence (except nicotine); other Axis I psychiatric disorders; medical and neurological disorders (hypertension, diabetes, stroke); history of eye trauma; or less than 7 years of formal education  Statistically controlled for years of education and nicotine dependence  Groups were matched on age and gender | SZ and BD had wider retinal venules and narrower retinal arterioles compared to controls  Wider retinal venules and narrower retinal arterioles in SZ and BD were associated with poorer working memory | Neither |
| Asanad et al. (2021) (69) | 58 SSD (n=45 SZ and n=13 SZA)  35 controls | 37.2 (12.3)  41.1 (15.2) | Not reported | SD-OCT (Cirrus HD-OCT 5000) | Excluded individuals with ophthalmologic conditions; a history of major neurological or unstable medical illness; or substance use within the last month or substance dependence within the last three months (except smoking and marijuana)  Statistically controlled for age, sex, hypertension, diabetes, potential ophthalmologic confounders (axial length and intraocular pressure), and antipsychotics | SSD group had significantly reduced pRNFL thickness compared to controls  No significant group differences found for retinal GCC indices | Neither |
| Ascaso et al. (2010) (70) | 10 SZ  10 controls | 39.2 (13.5); range: 50-88  39.5 (13.6), range: 24-64 | Not reported | TD-OCT (Stratus OCT) | Excluded individuals with posterior pole pathology such as macular degeneration or diabetic retinopathy; glaucoma suspect; media opacification such as cataract or vitreous hemorrhage that prevented ocular and OCT examination  Groups were matched on age and BCVA | SZ had reduced pRNFL thickness compared to controls  No significant group differences found for macular thickness, foveal thickness, or macular volume | Neither |
| Bozali & Yalinbias (2022) (71) | 57 SZ  57 controls | 37.2 (9.9)  36.8 (9.6) | Not reported | SD-OCT (RS-3000 Advance OCT) | Excluded individuals with a refractive error >±3.0 D; IOP ≥21 mmHg; ocular pathologies, including macular degeneration,  any retinopathies, optic neuropathies, glaucoma, or history of ocular trauma, or any ocular surgery that might interfere with the  OCT measurement; media opacity affecting the imaging; or systemic diseases or inflammatory diseases (e.g., diabetes mellitus)  Groups were matched on age, gender, and BCVA | No significant group difference found for central macular thickness  Mean outer retinal layer thickness and pRNFL thickness were reduced in SZ compared to controls | Neither |
| Carriello et al. (2024) (72) | 35 SZ  35 controls | 36.51 (12.41)  36.74 (11.94) | Not reported | SD-OCT (Spectralis HRA+OCT) | Excluded individuals with an ophthalmologic condition; IOP≥ 21 mm Hg; refractive error ≥ ±6 SPH D; intellectual disability; substance abuse; reported severe head trauma; uncontrolled medical condition; neurologic/rheumatologic/autoimmune condition; chronic infectious disease (e.g., HIV/AIDS, hepatitis); or nearsightedness exceeding 6 degrees  Statistically controlled for smoking status, clinical disease, and BMI  Groups were matched on age, sex, BMI, and smoking status | SZ had reduced macular volume and thickness compared to controls  No significant group differences in pRNFL or mRNFL thickness  Neither serum CRP nor IL-6 levels were associated with retinal thickness or volume  PANSS total, general, and positive symptom severity were positively associated with pRNFL thickness in SZ | Neither |
| Chu et al. (2012) (73) | 49 SSD (n=38 SZ and n=11 SZA)  40 controls | 29.9 (8.74)  29.5 (6.12) | 4.4 (3.6) | TD-OCT (Stratus OCT) | Excluded individuals with concurrent or history of systemic disease (e.g. diabetes or autoimmune disease) that could involve the eyes; a history of neurological or ophthalmological disease known to affect the visual pathway (e.g. glaucoma); high myopia (≥ ±6 D); previous head injury with loss of consciousness; or drug or alcohol dependence  Statistically controlled for age, gender, and illness duration  Groups were matched on age, gender, visual fields, and visual acuity | No significant differences found in whole retina pRNFL thickness or macular volume between SSD and controls  SZA had reduced right nasal quadrant pRNFL thickness compared to SZ  Reduced macular volume was associated with more severe positive symptoms | Neither |
| Daneshvar et al. (2023) (74) | 22 SZ on risperidone  Divided SZ into two subgroups:  1) n=8 with illness duration ≤2 years (DITYL)  2) n=14 with illness duration >2 years (DIMTY)  22 controls | 35.86 (9.29)  34.36 (6.56) | DITYL: ≤2 years  DIMTY: >2 years | SD-OCT/SD-OCTA (Optovue RTVue-XR Avanti OCT) | Excluded individuals taking the following medications: 4-aminoquinolines, amiodarone, calcium channel blockers; with alcohol consumption; with certain medical conditions that may affect the optic nerve head neurovasculature (uncontrolled hypertension, increased intracranial pressure, seizures); with a history of pregnancy; with a history of drug abuse; with a systemic illness; with ocular disease (e.g., glaucoma); or with intraocular surgery or trauma  Groups were matched on age, gender, cigarette smoking status, and IOP | Combined SZ group had higher cup-to-disc area, vertical cup-to-disc, and horizontal cup-to-disc ratios compared to controls  No other significant differences between SZ and controls were found for other optic nerve parameters (VDs, cup volume, pRNFL thickness)  DITYL and DIMTY did not differ on any optic nerve head variables  SZ with the most severe PANSS symptoms had increased VD compared to SZ with moderately severe symptoms | Neither |
| Friedel et al. (2022) (75) | 25 paranoid SZ  25 controls | 39.0 (12.0)  37.0 (12.0) | 9.0 (8.0) | SD-OCT (Spectralis OCT) | Excluded individuals with neurological diseases; ophthalmological diseases; myopia <−7 D or  hyperopia >+7 D; substance abuse; or diabetes mellitus  Groups were matched on age, sex, and smoking status | SZ had reduced ONL, GCL, and macular thickness compared to controls  No correlation between OCT indices and illness duration, PANSS scores, or CPZ found | Neither |
| Gandu et al. (2021) (76) | 30 SSD (SZ and SZA; n=10 early-course SSD and n=16 chronic SSD)  22 controls | 36.2 (12.7)  37.4 (11.5) | 12.49 (12.41) | SS-OCT, SD-OCT (Spectralis OCT, DRI Topcon Triton SS-OCT) | Excluded individuals with a history of substance dependence or abuse within the past 6 months; ophthalmologic condition; myopia >4.0 D; current pregnancy/breastfeeding; head injury with neurologic sequelae; intellectual disability; or neurologic disorder  Statistically controlled for cardiovascular and metabolic illness, BP, BMI, BCVA, and smoking status in the past 30 days  Groups were matched on age, sex, race, BCVA, cardiometabolic disease status, and BP | No significant group differences found for peripapillary or macular layer measures  Thinner macular layers and total retinal peripapillary layer were associated with poorer global functioning  Thinner peripapillary layers were associated with worse YMRS scores and poorer cognitive abilities | Neither |
| Kani et al. (2023) (77) | 24 SZ  9 controls | 33.83 (9.13)  32.23 (8.04) | 11.63 (7.57) | SD-OCT (Optovue RTVue OCT) | Excluded individuals with intellectual disability; alcohol or substance use disorder; history of head trauma leading to unconsciousness; degenerative neurological, immunological, or systemic disease that may affect the visual pathways; a primary ophthalmologic disease  (glaucoma, retinal disease, AMD, diabetic retinopathy, degenerative myopia); myopia, hyperopia, or astigmatism ≥1 D; or pathology such as cataract, corneal leukoma or vitreous hemorrhage that  may affect the ocular examination and OCT measurement  Groups were matched on age, gender, education level, monthly income, and smoking status | No significant group differences found for pRNFL thickness or GCL thickness  No significant correlations found between retinal thickness indices and age of onset of illness, illness duration, number of hospitalizations, or CPZ  More severe PANSS symptoms were correlated with increased pRNFL thickness  Positive correlations were found between pRNFL/GCL thickness and cognitive functioning | Neither |
| Koman-Wierdak et al. (2021) (78) | 12 SZ  8 BD  15 controls | 26.33 (5.26), range: 19-35  24.13 (8.85), range: 15-43  26.8 (SD not reported), range: 23-35 | 3.55 (3.50) | SD-OCT/OCTA (Optovue OCT) | Excluded individuals with a systemic disease (diabetes mellitus, cardiovascular disorders); alcohol and other psychoactive substance dependence; or ophthalmological condition that may affect OCT or OCTA measurements  Groups were matched on age and gender | SZ had significantly reduced VD in the macular deep vascular complex compared to BD and controls  SZ and BD had significantly reduced macular thickness in the whole vascular complex and fovea thickness compared to controls | Neither |
| Korann et al. (2022) (79) | 20 SZ  17 controls | 31.65 (5.62)  29.13 (5.16) | 7.37 (6.10) | 3nethra Classic (Non-mydriatic fundus camera with a 40-degree field view by Forus Health Pvt Itd) | Excluded individuals with comorbid substance abuse or dependence in the previous year (except for nicotine abuse or dependence); concomitant Axis-I mental illness; lifetime incidence of non-communicable diseases (hypertension, diabetes mellitus, stroke); ocular injuries; metallic implants in the body; left- or mixed-handedness; ECT treatment in the previous 6 months; or those who were currently pregnant or breastfeeding  Statistical controlled for age, sex, CPZ, serum creatinine levels, fasting blood glucose levels, and whole-brain volume  Groups were matched on age, gender, and biochemical parameters (blood urea, serum creatinine, and fasting glucose levels) | Retinal arterial tortuosity, tortuosity, and fractal dimension were associated with white matter lesions in SZ (but not in controls) | Neither |
| Kurhan et al. (2024) (80) | 30 FEP (SSD [specific diagnoses not reported]) | 36.2 (13.7) | Not reported | SD-OCT (Spectralis OCT) | Excluded individuals with abnormal IOP; corrected visual acuity < 20/32; ocular diseases (e.g., glaucoma, retinopathy); history of ocular surgery, systemic medication use that could affect retinal structures; any ocular opacity (e.g., cataracts or corneal opacity) interfering with OCT; refraction ≥±3 D SPH; any other psychiatric disorders; history of smoking, alcohol, or other substance use disorders; history of psychiatric treatment; currently receiving psychiatric treatment; medical comorbidities (e.g., diabetes, hypertension, or any physical illnesses); or those who were overweight or obese | FEP had significant increases in IPL, OPL, and total retinal thickness between their first psychotic episode and after they achieved remission through ECT  Age was negatively correlated with total retinal thickness  Pre-ECT PANSS-positive, general psychopathology, and total scores were positively correlated with pre-ECT RPE thickness | Neither |
| Li et al. (2022) (81) | 36 PSD (n=18 SZ, n=9 SZA, and n=5 BD with psychotic features)  20 controls | 34.5^[[7]](#footnote-7)^ (25.0-46.0)^[[8]](#footnote-8)^  34.0^7^ (28.0-47.0)^8^ | Not reported | SS-OCT (Topcon DRI OCT-1 Atlantis) | Excluded individuals with substance dependence within the past 6 months; glaucoma, macular degeneration; retinal vascular occlusions; ocular trauma; myopia >4.0 D; current pregnancy or breastfeeding; head injury with neurological sequelae; intellectual disability; or a history of neurologic disorders  Statistically controlled for age, race, BMI, visual acuity, illness duration, and CPZ  Groups were matched on age, sex, race, BMI, and BCVA | No significant differences found between PSD and controls for CT, CVD, or CVV  Male PSD CVD was reduced compared to male controls  Female PSD CT and CVV were reduced compared to female controls | Neither |
| Meier et al. (2013) (82) | 27 SZ  412 participants without SZ—divided them into comparison groups:  1) 110 with hypertension  2) 154 with pre-diabetes or diabetes  3) 210 with persistent tobacco dependence  4) 188 with persistent depression  Participants were from the Dunedin Study cohort | All participants received retinal imaging at age 38 | Not reported | Canon NMR-45 with a 20D SLR backing | Controlled for hypertension and diabetes by including those with these conditions as comparison groups  Also statistically controlled for systolic BP, diastolic BP, HbA1c, persistent tobacco dependence, persistent depression, antipsychotics | SZ had wider retinal venules than all groups except for hypertension group  Individuals who experienced greater psychotic symptoms at age 11 had wider retinal venules at age 38 | Neither |
| Munivenkatappa et al. (2025) (83) | 36 SZ  31 controls | 30.72 (6.07)  36.09 (6.72) | 4.97 (3.07) | SD-OCT (Spectralis OCT) | Excluded individuals with conditions like macular degeneration, glaucoma, recent  ocular surgery or ocular trauma; diabetes; hypertension; media opacities that preclude OCT examination; significantly impaired visual acuity; other comorbid psychiatric diagnoses (including substance use disorder); or <18 years old or >45 years old | SZ had increased macular CSF thickness, as well as reduced macular thickness and volume compared to controls  Symptom severity, illness duration, and drug naïve status were not significantly correlated with any OCT parameters | Neither |
| Shoham et al. (2023)^[[9]](#footnote-9)^ (84) | 159 SSD (F20-F29)  97,713 controls  From the UK Biobank | Entire sample: 58.0^[[10]](#footnote-10)^ (50-63)^[[11]](#footnote-11)^ | Not reported | SD-OCT | Statistically controlled for age, sex, SES, BMI, diabetes, and smoking status | No significant association found between OCT measures and subsequent psychotic experiences | Neither |
| Silverstein et al. (2018) (85) | 32 SZ divided into two subgroups:  1) n=21 without diabetes or hypertension (Sz-DH)  2) n=11 with diabetes or hypertension (Sz + DH)  32 controls divided into two subgroups:  1) n=21 without diabetes or hypertension (Con-DH)  2) n=11 with diabetes or hypertension (Con + DH) | 40.46 (12.09)  35.14 (11.07)  50.91 (5.32)  39.19 (11.03)  37.67 (9.07)  42.09 (14.1) | Not reported | SD-OCT (Cirrus HD-OCT 4000) | Excluded individuals with a(n) ophthalmologic condition/disease/injury; neurologic disorders; intellectual or developmental disorders (e.g., autism); active substance abuse disorders for at least 6 months; or a history of head injury with loss of consciousness of >10 minutes  Controlled for diabetes and hypertension via study design  SZ and controls were matched on age and gender | No significant group differences in pRNFL, macula, or GCL-IPL thickness  Reduced retinal thickness was related to the presence of diabetes/hypertension in entire sample  SZ had enlarged cup volume and CDR, which was unrelated to medical comorbidity, but was related to increased cognitive symptoms | Neither |
| Yilmaz et al. (2016) (86) | 34 SZ  30 controls | 39.85 (10.28)  38.59 (9.58) | Not reported | SD-OCT (Cirrus 4000 HD-OCT) | Excluded individuals with retinal, macular, or optic dis disease; prominent cataract; corneal pathology impairing measurement quality; retina laser or ocular surgery history; systemic disease such as hypertension or diabetes mellitus, that could affect macular or RNFL thickness; or smokers  Groups were matched on age and sex | pRNFL and macular thickness were reduced in SZ compared to controls | Neither |

*Note*. Criteria for categorization were as follows: Studies where the mean illness duration was less than 5 years and/or where early stage and later stage patients were directly compared and where the findings indicated a difference no greater than would be expected by normal aging and/or where extent of retinal thinning was not significantly correlated with illness duration were categorized as primarily supporting the Neurodevelopmental hypothesis. Studies where the mean illness duration was greater than 5 years and/or where early stage and later stage patients were directly compared and where the findings indicated greater retinal atrophy in the later stage group and/or where extent of retinal thinning was correlated with illness duration were categorized as primarily supporting the Neurodegenerative hypothesis. Studies that present evidence supporting both hypotheses are labeled as such. The fourth category used was “Neither,” for studies in which the findings did not have a direct bearing on the neurodegenerative, neurodevelopmental, or progressive neurodevelopmental models.

*Abbreviations*: AD, Alzheimer’s disease; AH, auditory hallucinations; AMD, age-related macular degeneration; BCVA, best-corrected visual acuity; BD, bipolar disorder; BMI, body mass index; BP, blood pressure; CFT, central foveal thickness; CHR, clinical high risk for psychosis; CNS, central nervous system; CNTF, ciliary neurotrophic factor; CPZ, chlorpromazine equivalent dose; CRAE, central retinal artery equivalent; CRP, C-reactive protein; CT, choroid thickness; CVD, choroid vascular density; CVI, choroidal vascularity index; CVV, choroid vascular volume; CYL, optical cylindrical equivalent; D, diopter; DCP, deep capillary plexuses; DD, delusional disorder; DDLS, Disc Damage Likelihood Scale (87); DUP, duration of untreated psychosis; ECT, electroconvulsive therapy; ERG, electroretinography; FAZ, foveal avascular zone; FEP, first-episode psychosis; GCC, ganglion cell complex; GMV, gray matter volume; HDL, high-density lipoprotein; HFL, Henle fiber layer; HbA1c, glycosylated hemoglobin; IL-6, interleukin 6; IOP, intraocular pressure; ISL, inner segment layer; LCA/SCA ratio, luminal choroidal area/stromal choroidal area ratio; macula CSF, macula central subfield; MAP, mean arterial pressure; MMSE, Mini-Mental State Examination (88); MS, multiple sclerosis; MZ, myoid zone; OCT, optical coherence tomography; ONL, outer nuclear layer; OPL, outer plexiform layer; PANSS, the Positive and Negative Syndrome Scale (89); PD, perfusion density; PRS, polygenic risk score; PSD, psychotic spectrum disorder; RAG, retinal age gap; RATI, retinal arteriolar tortuosity index; RCP, retinal capillary plexuses (both superior capillary plexuses and deep capillary plexuses); RNFL, retinal nerve fiber layer; RPC, radial peripapillary capillaries; RPE, retinal pigment epithelium; RVTI, retinal venular tortuosity index; SCP, superior capillary plexuses; SNPs, single nucleotide polymorphisms; SPD, schizotypal personality disorder; SPH, optical spherical equivalent; SSD, schizophrenia spectrum disorder; SVD, superficial vessel density; SZ, schizophrenia; TNF-α, tumor necrosis factor-alpha; VD, vessel density; VH, visual hallucinations; YMRS, Young Mania Rating Scale (90)

**References**

1. Akin F, Danaci AE, Kayikcioglu RO, Tasci MY. Retinal abnormalities and their relationship with social cognition in patients with schizophrenia and their healthy siblings. Dusunen Adam Journal of Psychiatry and Neurological Sciences (2024) 37(4):179-88. doi: 10.14744/DAJPNS.2024.00259.

2. Kerr SL, Neale JM. Emotion perception in schizophrenia: specific deficit or further evidence of generalized poor performance? J Abnorm Psychol (1993) 102(2):312-8. doi: 10.1037//0021-843x.102.2.312.

3. Bannai D, Adhan I, Katz R, Kim LA, Keshavan M, Miller JB, et al. Quantifying retinal microvascular morphology in schizophrenia using swept-source optical coherence tomography angiography. Schizophrenia Bulletin (2022) 48(1):80-9. doi: 10.1093/schbul/sbab111.

4. Bagci KA, Memis PN, Akhoroz M, Karakilinc BNT, Cop E. Exploring retinal thickness variations in adolescents with first episode psychosis and schizophrenia: A comparative study with healthy siblings and controls. Psychiatry Res Neuroimaging (2025) 352:112020. Epub 20250630. doi: 10.1016/j.pscychresns.2025.112020.

5. Boudriot E, Stephan M, Rabe F, Smigielski L, Schmitt A, Falkai P, et al. Genetic Analysis of Retinal Cell Types in Neuropsychiatric Disorders. JAMA Psychiatry (2025) 82(3):285-95. doi: 10.1001/jamapsychiatry.2024.4230.

6. Demirlek C, Atas F, Yalincetin B, Gurbuz MS, Cesim E, Demir M, et al. Choroidal structural analysis in ultra-high risk and first-episode psychosis. European Neuropsychopharmacology (2023) 70:72-80. Epub 20230315. doi: 10.1016/j.euroneuro.2023.02.016.

7. Demirlek C, Arslan B, Eyuboglu MS, Yalincetin B, Atas F, Cesim E, et al. Retina in clinical high-risk and first-episode psychosis. Schizophrenia Bulletin (2024). Epub 20241102. doi: 10.1093/schbul/sbae189.

8. Fuyi Q, Xiang C, Xinling Z, Zeyi G, Liu Y, Jia W, et al. Association between retinal nerve fiber layer thickness and psychiatric disorders: A mendelian randomization study. BMC Psychiatry (2024) 24(1):640. Epub 20240930. doi: 10.1186/s12888-024-06100-8.

9. Gonzalez-Diaz JM, Sanchez Dalmau B, Camos-Carreras A, Alba-Arbalat S, Amoretti S, Forte MF, et al. Retinal structure and its relationship with premorbid, clinical, and cognitive variables in young Spanish patients with early course schizophrenia spectrum disorders. Eur Neuropsychopharmacol (2025) 92:38-47. Epub 20241222. doi: 10.1016/j.euroneuro.2024.12.006.

10. Huang J, Song X, Xu Y, Wang L, Li Y, Tian H, et al. Reliability and diagnostic validity of a novel visual disturbance subjective experience scale in Chinese patients with schizophrenia. Psychiatry and Clinical Psychopharmacology (2020) 30(3):307-12. doi: 10.5455/PCP.20200302022126.

11. Kaya H, Ayik B, Tasdelen R, Sevimli N, Ertekin E. Comparing retinal changes measured by optical coherence tomography in patients with schizophrenia and their siblings with healthy controls: Are retinal findings potential endophenotype candidates? Asian Journal of Psychiatry (2022) 72:103089. Epub 20220325. doi: 10.1016/j.ajp.2022.103089.

12. Kurtulmus A, Elbay A, Parlakkaya FB, Kilicarslan T, Ozdemir MH, Kirpinar I. An investigation of retinal layer thicknesses in unaffected first-degree relatives of schizophrenia patients. Schizophrenia Research (2020) 218:255-61. Epub 20200114. doi: 10.1016/j.schres.2019.12.034.

13. Meier MH, Gillespie NA, Hansell NK, Hewitt AW, Hickie IB, Lu Y, et al. Retinal microvessels reflect familial vulnerability to psychotic symptoms: A comparison of twins discordant for psychotic symptoms and controls. Schizophrenia Research (2015) 164(1-3):47-52. Epub 20150216. doi: 10.1016/j.schres.2015.01.045.

14. Gillespie NA, Henders AK, Davenport TA, Hermens DF, Wright MJ, Martin NG, et al. The Brisbane Longitudinal Twin Study: Pathways to cannabis use, abuse, and dependence project-current status, preliminary results, and future directions. Twin Research and Human Genetics (2013) 16(1):21-33. Epub 20121128. doi: 10.1017/thg.2012.111.

15. Wright MJ, Martin NG. Brisbane Adolescent Twin Study: Outline of study methods and research projects. Australian Journal of Psychology (2004) 56(2):65-78. doi: 10.1080/00049530410001734865.

16. Sarkar S, Rajalakshmi AR, Avudaiappan S, Eswaran S. Exploring the role of macular thickness as a potential early biomarker of neurodegeneration in acute schizophrenia. International Ophthalmology (2021) 41(8):2737-46. Epub 20210415. doi: 10.1007/s10792-021-01831-z.

17. Silverstein SM, Lai A, Green KM, Crosta C, Fradkin SI, Ramchandran RS. Retinal microvasculature in schizophrenia. Eye and Brain (2021) 13:205-17. Epub 20210724. doi: 10.2147/EB.S317186.

18. Zhuo C, Xiao B, Chen C, Jiang D, Li G, Ma X, et al. Abberant inverted U-shaped brain pattern and trait-related retinal impairment in schizophrenia patients with combined auditory and visual hallucinations: a pilot study. Brain Imaging Behav (2021) 15(2):738-47. doi: 10.1007/s11682-020-00281-y.

19. Zhuo C, Xiao B, Ji F, Lin X, Jiang D, Tian H, et al. Patients with first-episode untreated schizophrenia who experience concomitant visual disturbances and auditory hallucinations exhibit co-impairment of the brain and retinas-a pilot study. Brain Imaging Behav (2021) 15(3):1533-41. doi: 10.1007/s11682-020-00351-1.

20. Blose BA, Silverstein SM, Stuart KV, Keane PA, Khawaja AP, Wagner SK. Association between polygenic risk for schizophrenia and retinal morphology: A cross-sectional analysis of the United Kingdom Biobank. Psychiatry Research (2024) 339:116106. doi: <https://doi.org/10.1016/j.psychres.2024.116106>.

21. Boudriot E, Gabriel V, Popovic D, Pingen P, Yakimov V, Papiol S, et al. Signature of altered retinal microstructures and electrophysiology in schizophrenia spectrum disorders is associated with disease severity and polygenic risk. Biological Psychiatry (2024) 96(10):792-803. Epub 20240427. doi: 10.1016/j.biopsych.2024.04.014.

22. Fradkin SI, Bannai D, Lizano P, Lai A, Crosta C, Thompson JL, et al. Deep retinal layer microvasculature alterations in schizophrenia. Biomarkers in Neuropsychiatry (2024) 10:100084. doi: 10.1016/j.bionps.2024.100084.

23. Hosak L, Zeman T, Studnicka J, Stepanov A, Ustohal L, Michalec M, et al. Retinal arteriolar and venular diameters are widened in patients with schizophrenia. Psychiatry Clin Neurosci (2020) 74(11):619-21. Epub 20200912. doi: 10.1111/pcn.13123.

24. Hosak L, Sadykov E, Zapletalova J, Hosakova J, Stepanov A, Latalova K, et al. Widened retinal arteriolar and venular diameters are not an endophenotype of schizophrenia: A one-time cross-sectional study. Neuro Endocrinol Lett (2023) 44(5):290-6.

25. Krukow P, Domagala A, Silverstein SM. Specific association between retinal neural layer thinning and neurological soft signs in schizophrenia. European Archives of Psychiatry and Clinical Neuroscience (2024) 274(5):1237-40. Epub 20240120. doi: 10.1007/s00406-023-01742-3.

26. Liu Y, Huang L, Chen J, Tan S, Zhao K, Yan S, et al. Retinal venule correlation with schizophrenia. International Journal of Clinical and Experimental Medicine (2020) 13(9):6927-35.

27. Rabe F, Smigielski L, Georgiadis F, Kallen N, Omlor W, Edkins V, et al. Genetic susceptibility to schizophrenia through neuroinflammatory pathways associated with retinal thinness. Nat Ment Health (2025) 3(5):538-47. Epub 20250421. doi: 10.1038/s44220-025-00414-6.

28. Zhuo C, Ji F, Xiao B, Lin X, Chen C, Jiang D, et al. Antipsychotic agent-induced deterioration of the visual system in first-episode untreated patients with schizophrenia maybe self-limited: Findings from a secondary small sample follow-up study based on a pilot follow-up study. Psychiatry Research (2020) 286:112906. Epub 20200301. doi: 10.1016/j.psychres.2020.112906.

29. Zhuo C, Xiao B, Chen C, Jiang D, Li G, Ma X, et al. Antipsychotic agents deteriorate brain and retinal function in schizophrenia patients with combined auditory and visual hallucinations: A pilot study and secondary follow-up study. Brain Behav (2020) 10(6):e01611. Epub 20200414. doi: 10.1002/brb3.1611.

30. Alizadeh M, Delborde Y, Ahmadpanah M, Seifrabiee MA, Jahangard L, Bazzazi N, et al. Non-linear associations between retinal nerve fibre layer (RNFL) and positive and negative symptoms among men with acute and chronic schizophrenia spectrum disorder. J Psychiatr Res (2021) 141:81-91. Epub 20210607. doi: 10.1016/j.jpsychires.2021.06.007.

31. Antaki F, Kerexeta-Sarriegi J, Reis APR, Zhu Z, Chen R, Hu W, et al. The association of retinal age gap with schizophrenia: a cross-sectional analysis. Schizophr Res (2025) 283:180-7. Epub 20250724. doi: 10.1016/j.schres.2025.07.018.

32. Appaji A, Nagendra B, Chako DM, Padmanabha A, Hiremath CV, Jacob A, et al. Retinal vascular abnormalities in schizophrenia and bipolar disorder: A window to the brain. Bipolar Disord (2019) 21(7):634-41. Epub 20190508. doi: 10.1111/bdi.12779.

33. Appaji A, Nagendra B, Chako DM, Padmanabha A, Jacob A, Hiremath CV, et al. Examination of retinal vascular trajectory in schizophrenia and bipolar disorder. Psychiatry Clin Neurosci (2019) 73(12):738-44. Epub 20190911. doi: 10.1111/pcn.12921.

34. Appaji A, Nagendra B, Chako DM, Padmanabha A, Jacob A, Hiremath CV, et al. Retinal vascular tortuosity in schizophrenia and bipolar disorder. Schizophrenia Research (2019b) 212:26-32. Epub 20190826. doi: 10.1016/j.schres.2019.08.020.

35. Ascaso FJ, Rodriguez-Jimenez R, Cabezon L, Lopez-Anton R, Santabarbara J, De la Camara C, et al. Retinal nerve fiber layer and macular thickness in patients with schizophrenia: Influence of recent illness episodes. Psychiatry Research (2015) 229(1-2):230-6. Epub 20150715. doi: 10.1016/j.psychres.2015.07.028.

36. Bannai D, Lizano P, Kasetty M, Lutz O, Zeng V, Sarvode S, et al. Retinal layer abnormalities and their association with clinical and brain measures in psychotic disorders: A preliminary study. Psychiatry Research: Neuroimaging (2020) 299:111061. Epub 20200229. doi: 10.1016/j.pscychresns.2020.111061.

37. Blose BA, Lai A, Crosta C, Thompson JL, Silverstein SM. Retinal neurodegeneration as a potential biomarker of accelerated aging in schizophrenia spectrum disorders. Schizophrenia Bulletin (2023) 49(5):1316-24. doi: 10.1093/schbul/sbad102.

38. Boudriot E, Schworm B, Slapakova L, Hanken K, Jager I, Stephan M, et al. Optical coherence tomography reveals retinal thinning in schizophrenia spectrum disorders. European Archives of Psychiatry and Clinical Neuroscience (2023) 273(3):575-88. Epub 20220805. doi: 10.1007/s00406-022-01455-z.

39. Budakoglu O, Ozdemir K, Safak Y, Sen E, Taskale B. Retinal nerve fibre layer and peripapillary vascular density by optical coherence tomography angiography in schizophrenia. Clinical and Experimental Optometry (2021) 104(7):788-94. doi: 10.1080/08164622.2021.1878816.

40. Celik M, Kalenderoglu A, Sevgi Karadag A, Bekir Egilmez O, Han-Almis B, Simsek A. Decreases in ganglion cell layer and inner plexiform layer volumes correlate better with disease severity in schizophrenia patients than retinal nerve fiber layer thickness: Findings from spectral optic coherence tomography. European Psychiatry (2016) 32:9-15. Epub 20160120. doi: 10.1016/j.eurpsy.2015.10.006.

41. Delıbaş DH, Karti Ö, Erdoğan E, Şahin T, Bilgiç Ö, Erol A. Decreases in retinal nerve fiber layer and ganglion cell-inner plexiform layer thickness in schizophrenia, relation to insight: A controlled study. Anatolian Journal of Psychiatry (2018) 19(3):264-73. doi: 10.5455/apd.276720.

42. Domagala A, Domagala L, Kopis-Posiej N, Harciarek M, Krukow P. Differentiation of the retinal morphology aging trajectories in schizophrenia and their associations with cognitive dysfunctions. Front Psychiatry (2023) 14:1207608. Epub 20230719. doi: 10.3389/fpsyt.2023.1207608.

43. Hanifi Kokaçya M, Idil Çakmak A. Optical coherence tomography angiography in schizophrenia. Alpha Psychiatry (2022) 23(5):253-61. doi: 10.5152/alphapsychiatry.2022.21629.

44. Jerotic S, Ristic I, Pejovic S, Mihaljevic M, Pavlovic Z, Britvic D, et al. Retinal structural abnormalities in young adults with psychosis spectrum disorders. Progress in Neuro-Psychopharmacology & Biological Psychiatry (2020) 98:109825. Epub 20191121. doi: 10.1016/j.pnpbp.2019.109825.

45. Jerotic S, Lalovic N, Pejovic S, Mihaljevic M, Pavlovic Z, Britvic D, et al. Sex differences in macular thickness of the retina in patients with psychosis spectrum disorders. Prog Neuropsychopharmacol Biol Psychiatry (2021) 110:110280. Epub 20210207. doi: 10.1016/j.pnpbp.2021.110280.

46. Joe P, Ahmad M, Riley G, Weissman J, Smith RT, Malaspina D. A pilot study assessing retinal pathology in psychosis using optical coherence tomography: Choroidal and macular thickness. Psychiatry Res (2018) 263:158-61. Epub 20180306. doi: 10.1016/j.psychres.2018.03.011.

47. Kango A, Grover S, Gupta V, Sahoo S, Nehra R. A comparative study of retinal layer changes among patients with schizophrenia and healthy controls. Acta Neuropsychiatrica (2023) 35(3):165-76. Epub 20221207. doi: 10.1017/neu.2022.35.

48. Khalil DH, Aziz K, Khalil M, Khowyled A. Optical coherence tomography in Egyptian schizophrenics and its correlation to disease parameters. Delta Journal of Ophthalmology (2022) 23(3):198-205. doi: 10.1353/djo.djo_74_21.

49. Kozub KE, Shelepin IE, Chomskii AN, Sharybin EA, Ivanova EA. A structural and functiona study of the retina in patients with schizophrenia. Journal of Ophthalmology (Ukraine) (2020) 4(495):38-44.

50. Krukow P, Domagala A, Kiersztyn A, Blose BA, Lai A, Silverstein SM. The Retinal Age Gap as a Marker of Accelerated Aging in the Early Course of Schizophrenia. Schizophr Bull (2025). Epub 20250414. doi: 10.1093/schbul/sbaf038.

51. Kurt A, Ramazan Zor K, Kucuk E, Yildirim G, Erdal Ersan E. An Optical Coherence Tomography Study that Supports the Neurovascular Basis of Schizophrenia Disease. Alpha Psychiatry (2022) 23(1):12-7. Epub 20211230. doi: 10.1530/alphapsychiatry.2021.21207.

52. Kurtulmus A, Sahbaz C, Elbay A, Guler EM, Sonmez Avaroglu G, Kocyigit A, et al. Clinical and biological correlates of optical coherence tomography findings in schizophrenia. European Archives of Psychiatry and Clinical Neuroscience (2023) 273(8):1837-50. Epub 20230406. doi: 10.1007/s00406-023-01587-w.

53. Lai A, Crosta C, Loftin M, Silverstein SM. Retinal structural alterations in chronic versus first episode schizophrenia spectrum disorders. Biomarkers in Neuropsychiatry (2020) 2:100013. doi: 10.1016/j.bionps.2020.100013.

54. Lee WW, Tajunisah I, Sharmilla K, Peyman M, Subrayan V. Retinal nerve fiber layer structure abnormalities in schizophrenia and its relationship to disease state: Evidence from optical coherence tomography. Investigative Ophthalmology & Visual Science (2013) 54(12):7785-92. Epub 20131121. doi: 10.1167/iovs.13-12534.

55. Liu Y, Huang L, Tong Y, Chen J, Gao D, Yang F. Association of retinal nerve fiber abnormalities with serum CNTF and cognitive functions in schizophrenia patients. PeerJ (2020) 8:e9279. Epub 20200602. doi: 10.7717/peerj.9279.

56. Liu Y, Chen J, Huang L, Yan S, Bian Q, Yang F. Relationships among retinal nerve fiber layer thickness, vascular endothelial growth factor, and cognitive impairment in patients with schizophrenia. Neuropsychiatric Disease and Treatment (2021) 17:3597-606. Epub 20211209. doi: 10.2147/NDT.S336077.

57. Miller M, Zemon V, Nolan-Kenney R, Balcer LJ, Goff DC, Worthington M, et al. Optical coherence tomography of the retina in schizophrenia: Inter-device agreement and relations with perceptual function. Schizophr Res (2020) 219:13-8. Epub 20200111. doi: 10.1016/j.schres.2019.10.046.

58. Mota M, Pêgo P, Klut C, Coutinho I, Santos C, Pires G, et al. Evaluation of structural changes in the retina of patients with schizophrenia. Ophthalmology Research: An International Journal (2015) 4(2):45-52. doi: 10.9734/OR/2015/17953.

59. Murav’eva SV, Kozub KE, Pronin SV. Optical and electrophysiological techniques for functional assessment of vision system neuronal networks. Journal of Optical Technology (2021) 88(12):42-9. doi: 10.1364/JOT.88.000710.

60. Orum MH, Bulut M, Karadag AS, Dumlupinar E, Kalenderoglu A. Comparison of OCT findings of schizophrenia patients using FGA, clozapine, and SGA other than clozapine. Archives of Clinical Psychiatry (2020) 47(6):165-75. doi: 10.15761/0101-60830000000257.

61. Padmanabhan A, Prabhu PB, Vidyadharan V, Tharayil HM. Retinal Nerve Fiber Layer Thickness in Patients with Schizophrenia and Its Relation with Cognitive Impairment. Indian J Psychol Med (2024) 46(3):238-44. Epub 20240131. doi: 10.1177/02537176231223311.

62. Samani NN, Proudlock FA, Siram V, Suraweera C, Hutchinson C, Nelson CP, et al. Retinal layer abnormalities as biomarkers of schizophrenia. Schizophrenia Bulletin (2018) 44(4):876-85. doi: 10.1093/schbul/sbx130.

63. Schönfeldt-Lecuona C, Kregel T, Schmidt A, Kassubek J, Dreyhaupt J, Freudenmann RW, et al. Retinal single-layer analysis with optical coherence tomography (OCT) in schizophrenia spectrum disorder. Schizophrenia Research (2020) 219:5-12. Epub 20190411. doi: 10.1016/j.schres.2019.03.022.

64. Tasdelen R, Ayik B, Kaya H, Sevimli N. Investigation of the relationship between cognitive functions and retinal findings from spectral optical coherence tomography in patients with schizophrenia and their healthy siblings. Psychiatry Investigation (2023) 20(3):236-44. Epub 20230322. doi: 10.30773/pi.2022.0268.

65. Topcu-Yilmaz P, Aydin M, Ilhan BC. Evaluation of retinal nerve fiber layer, macular, and choroidal thickness in schizophrenia: Spectral optic coherence tomography findings. Psychiatry and Clinical Psychopharmacology (2019) 29(1):28-33. doi: 10.1080/24750573.2018.1426693.

66. Wagner SK, Cortina-Borja M, Silverstein SM, Zhou Y, Romero-Bascones D, Struyven RR, et al. Association between retinal features from multimodal imaging and schizophrenia. JAMA Psychiatry (2023) 80(5):478-87. doi: 10.1001/jamapsychiatry.2023.0171.

67. Altun IK, Turedi N, Aras N, Atagun MI. Psychopharmacological Signatures in the Retina in Schizophrenia and Bipolar Disorder: An Optic Coherence Tomography Study. Psychiatr Danub (2020) 32(3-4):351-8. doi: 10.24869/psyd.2020.351.

68. Appaji A, Nagendra B, Chako DM, Padmanabha A, Jacob A, Hiremath CV, et al. Relation between retinal vascular abnormalities and working memory impairment in patients with schizophrenia and bipolar disorder. Asian J Psychiatr (2020) 49:101942. Epub 20200210. doi: 10.1016/j.ajp.2020.101942.

69. Asanad S, O'Neill H, Addis H, Chen S, Wang J, Goldwaser E, et al. Neuroretinal Biomarkers for Schizophrenia Spectrum Disorders. Transl Vis Sci Technol (2021) 10(4):29. doi: 10.1167/tvst.10.4.29.

70. Ascaso FJ, Cabezón L, Quintanilla MÁ, Gutiérrez Galve L, López-Antón R, Cristóbal JA, et al. Retinal nerve fiber layer thickness measured by optical coherence tomography in patients with schizophrenia: A short report. The European Journal of Psychiatry (2010) 24(4):227-35. doi: 10.4321/S0213-61632010000400005.

71. Bozali E, Yalinbas D. Analysis of the Thickness of the Outer Retinal Layer Using Optical Coherence Tomography - A Predictor of Visual Acuity in Schizophrenia. Klin Monbl Augenheilkd (2022) 239(10):1232-8. Epub 20220323. doi: 10.1055/a-1741-7988.

72. Carriello MA, Costa DFB, Alvim PHP, Pestana MC, Bicudo DDS, Gomes EMP, et al. Retinal layers and symptoms and inflammation in schizophrenia. Eur Arch Psychiatry Clin Neurosci (2024) 274(5):1115-24. Epub 20230316. doi: 10.1007/s00406-023-01583-0.

73. Chu EM, Kolappan M, Barnes TR, Joyce EM, Ron MA. A window into the brain: an in vivo study of the retina in schizophrenia using optical coherence tomography. Psychiatry Res (2012) 203(1):89-94. Epub 20120820. doi: 10.1016/j.pscychresns.2011.08.011.

74. Daneshvar R, Naghib M, Fayyazi Bordbar MR, Faridhosseini F, Fotouhi M, Motamed Shariati M. Optic nerve head neurovascular assessments in patients with schizophrenia: A cross-sectional study. Health Sci Rep (2024) 7(5):e2100. Epub 20240508. doi: 10.1002/hsr2.2100.

75. Friedel EBN, Hahn HT, Maier S, Kuchlin S, Reich M, Runge K, et al. Structural and functional retinal alterations in patients with paranoid schizophrenia. Transl Psychiatry (2022) 12(1):402. Epub 20220923. doi: 10.1038/s41398-022-02167-7.

76. Gandu S, Bannai D, Adhan I, Kasetty M, Katz R, Zang R, et al. Inter-device reliability of swept source and spectral domain optical coherence tomography and retinal layer differences in schizophrenia. Biomarkers in Neuropsychiatry (2021) 5:100036. doi: 10.1016/j.bionps.2021.100036.

77. Kani AS, Çam CS, Çelik EB, Dural U, Dönmez MD, Turhan SA, et al. Neuropsychological and clinical correlations of optical coherenec tomography findings in patients with schizophrenia. Clinical and Experimental Health Sciences (2023) 13:739-47. doi: 10.33808/clinexphealthsci.1331234.

78. Koman-Wierdak E, Rog J, Brzozowska A, Toro MD, Bonfiglio V, Zaluska-Ogryzek K, et al. Analysis of the Peripapillary and Macular Regions Using OCT Angiography in Patients with Schizophrenia and Bipolar Disorder. J Clin Med (2021) 10(18). Epub 20210913. doi: 10.3390/jcm10184131.

79. Korann V, Suhas S, Appaji A, Nagendra B, Padmanabha A, Jacob A, et al. Association between retinal vascular measures and brain white matter lesions in schizophrenia. Asian Journal of Psychiatry (2022) 70:103042. Epub 20220219. doi: 10.1016/j.ajp.2022.103042.

80. Kurhan F, Yildiz V, Kamis GZ, Karatas K, Batur M. Evaluation of the Electroconvulsive Therapy's Impact on Retinal Structures in First-Episode Psychosis Patients Using Optical Coherence Tomography. Schizophr Bull (2024). Epub 20241126. doi: 10.1093/schbul/sbae187.

81. Li CY, Garg I, Bannai D, Kasetty M, Katz R, Adhan I, et al. Sex-Specific Changes in Choroid Vasculature Among Patients with Schizophrenia and Bipolar Disorder. Clin Ophthalmol (2022) 16:2363-71. Epub 20220728. doi: 10.2147/OPTH.S352731.

82. Meier MH, Shalev I, Moffitt TE, Kapur S, Keefe RS, Wong TY, et al. Microvascular abnormality in schizophrenia as shown by retinal imaging. Am J Psychiatry (2013) 170(12):1451-9. doi: 10.1176/appi.ajp.2013.13020234.

83. Munivenkatappa S, Ganne P, Parri M, U S, Avula VCR. Optical coherence tomography in patients with schizophrenia: An exploratory study. Psychiatry Res Neuroimaging (2025) 349:111972. Epub 20250305. doi: 10.1016/j.pscychresns.2025.111972.

84. Shoham N, Lewis G, Hayes JF, Silverstein SM, Cooper C. Association between visual impairment and psychosis: A longitudinal study and nested case-control study of adults. Schizophr Res (2023) 254:81-9. Epub 20230217. doi: 10.1016/j.schres.2023.02.017.

85. Silverstein SM, Paterno D, Cherneski L, Green S. Optical coherence tomography indices of structural retinal pathology in schizophrenia. Psychological Medicine (2018) 48(12):2023-33. Epub 20171213. doi: 10.1017/S0033291717003555.

86. Yilmaz U, Kucuk E, Ulgen A, Ozkose A, Demircan S, Ulusoy DM, et al. Retinal nerve fiber layer and macular thickness measurement in patients with schizophrenia. Eur J Ophthalmol (2016) 26(4):375-8. Epub 20151229. doi: 10.5301/ejo.5000723.

87. Spaeth GL, Henderer J, Liu C, Kesen M, Altangerel U, Bayer A, et al. The disc damage likelihood scale: reproducibility of a new method of estimating the amount of optic nerve damage caused by glaucoma. Trans Am Ophthalmol Soc (2002) 100:181-5; discussion 5-6.

88. Folstein MF, Folstein SE, McHugh PR. "Mini-mental state". A practical method for grading the cognitive state of patients for the clinician. J Psychiatr Res (1975) 12(3):189-98. doi: 10.1016/0022-3956(75)90026-6.

89. Kay SR, Fiszbein A, Opler LA. The positive and negative syndrome scale (PANSS) for schizophrenia. Schizophr Bull (1987) 13(2):261-76. doi: 10.1093/schbul/13.2.261.

90. Young RC, Biggs JT, Ziegler VE, Meyer DA. A rating scale for mania: reliability, validity and sensitivity. Br J Psychiatry (1978) 133:429-35. doi: 10.1192/bjp.133.5.429.

1. Median [↑](#footnote-ref-1)
2. Interquartile range [↑](#footnote-ref-2)
3. Mendelian randomization study [↑](#footnote-ref-3)
4. While total macula and specific layer thinning were related to poorer cognition in this established illness sample, longer illness duration was correlated with increased thickness [↑](#footnote-ref-4)
5. Median [↑](#footnote-ref-5)
6. Interquartile range [↑](#footnote-ref-6)
7. Median [↑](#footnote-ref-7)
8. Interquartile range [↑](#footnote-ref-8)
9. Nested case-control study and cohort study [↑](#footnote-ref-9)
10. Median [↑](#footnote-ref-10)
11. Interquartile range [↑](#footnote-ref-11)
